# Functional Networks in Developmental Dyslexia: Auditory Discrimination of Words and Pseudowords

**DOI:** 10.3390/neurosci7010021

**Published:** 2026-02-03

**Authors:** Tihomir Taskov, Juliana Dushanova

**Affiliations:** 1 Department of Physiology and Pathophysiology, Faculty of Medicine, Medical University of Sofia, 1431 Sofia, Bulgaria; t.taskov@abv.bg; 2 Institute of Neurobiology, Bulgarian Academy of Sciences, 1113 Sofia, Bulgaria

**Keywords:** developmental dyslexia, listening, functional connectivity, discrimination of words and pseudowords

## Abstract

Developmental dyslexia (DD) often involves difficulties in phonological processing of speech. Objectives: While underlying neural changes have been identified in terms of stimulus- and task-related responses within specific brain regions and their neural connectivity, there is still limited understanding of how these changes affect the overall organization of brain networks. Methods: This study used EEG and functional network analysis, focusing on small-world propensity across various frequency bands (from δ to γ), to explore the global brain organization during the auditory discrimination of words and pseudowords in children with DD. Results: The main finding revealed a systemic inefficiency in the functional network of individuals with DD, which did not achieve the optimal small-world propensity. This inefficiency arises from a fundamental trade-off between localized specialization and global communication. During word listening, the δ-/γ1-networks (related to impaired syllabic and phonemic processing of words) and the θ-/β-networks (related to pseudoword listening) in the DD group showed lower local clustering and connectivity compared to the control group, resulting in reduced functional segregation. In particular, the θ-/β-networks for words in the DD group exhibited a less optimal balance between specialized local processing and effective global communication. Centralized midline hubs, such as the postcentral gyrus (PstCG) and inferior frontal gyrus (IFG), which are crucial for global coordination, attention, and executive control, were either absent or inconsistent in individuals with DD. Consequently, the DD network adopted a constrained, motor-compensatory, and left-lateralized strategy. This led to the redirection of information flow and processing effort toward the left PstCG/IFG loop, interpreted as a compensatory effort to counteract automatic processing failures. Additionally, the γ1-network, which is involved in phonetic feature binding, lacked engagement from posterior sensory hubs, forcing this critical process into a slow and effortful motor loop. The γ2-network exhibited unusual activation of right-hemisphere posterior areas during word processing, while it employed a simpler, less mature routing strategy for pseudoword listening, which further diminished global communication. Conclusions: This functionality highlights the core phonological and temporal processing deficits characteristic of dyslexia.

## 1. Introduction

### 1.1. Developmental Dyslexia: The Phonological Deficit and Cognitive Models

Developmental Dyslexia (DD) is a specific learning disorder characterized by significant difficulties in acquiring reading and writing skills, despite normal scores on general intelligence tests [1,2]. Developmental Dyslexia is characterized by unexpected difficulty in reading, spelling, and decoding, primarily resulting from a deficit in the phonological component of language [3,4]. A foundational aspect of this deficit is often traced back to the inability to accurately and efficiently process rapidly changing auditory stimuli, which underpins the ability to distinguish and sequence the phonemic units that constitute words and pseudowords [5,6]. Understanding the neural mechanisms underlying this core deficit is crucial, as it dictates the efficacy of rehabilitation and educational strategies [7,8]. The varied linguistic and cognitive deficits observed in dyslexia are often explained by cognitive models, such as the dual-route model of reading [9]. The dual-route model proposes two primary ways for reading words. The lexical route is used for familiar words. The word’s visual form is recognized automatically as a member of the mental lexicon and directly linked to its verbal semantic representation. The sublexical route is used for unfamiliar words and pseudo-words. The word is broken down into its constituent letters and corresponding phonemes based on grapheme-to-phoneme correspondence rules [10,11]. The impairment in either route leads to distinct reading difficulty patterns. In phonological dyslexia (impaired sublexical pathway), children have difficulty converting letters into sounds. They tend to use the intact lexical route, resulting in good reading of irregular words (which are recognized visually) but poor reading of pseudo-words (which require the sublexical route). In surface dyslexia (impaired lexical route), children rely on the sublexical route. They can often process pseudo-words but have difficulty with irregular words because their direct visual-semantic link is impaired [10,11].

Developmental dyslexia is heterogeneous, involving a variety of deficits that can occur independently or in combination [12,13]. In phonological deficits, the problems are with processing the sound structure of language [14,15]. Visual magnocellular deficits are based on dysfunction in the visual pathway that mediates motion perception and object localization, linked to the dorsal visual stream [16,17]. Difficulties with attentional deficits are related to orienting and focusing visual-spatial attention [18]. The double deficit hypothesis further categorizes dyslexic types based on two separate sources of disability [19,20]. The phonological type experiences difficulty with tasks requiring phonological awareness, such as phoneme segmentation and sound blending [14,21]. The naming speed deficit type has problems with the rapid recognition and retrieval of visually presented language stimuli, resulting in slow and inaccurate word identification [22]. The combined deficits group exhibits both phonological and naming-speed deficits [19].

### 1.2. Neurobiological Foundations: Streams and Atypical Neural Correlates

Neuropsychological evidence suggests that distinct neural pathways, the dorsal and ventral routes, underlie sublexical and lexical processes, respectively [23,24]. The ventral (lexical) stream primarily involves anterior, superior, and middle temporal lobe regions such as the occipitotemporal area [25]. It is thought to be involved in processing speech for comprehension and is bilaterally organized [24]. The neuroanatomical correlates include the left basal temporal language area, the posterior middle temporal gyrus, and the inferior frontal gyrus (IFG), which act as a crucial route for the lexico-semantic pathway of speech comprehension [26]. Dorsal (sublexical) stream involves the boundary of the temporal and parietal lobes, including the posterior planum temporale and posterior frontal lobe [27,28]. It plays a major role in directing visual attention, controlling eye movements for reading, and translating acoustic speech signals into articulatory representations [29]. This stream is typically strongly left-dominant [30]. Neuroanatomical correlates are the left-lateralized superior temporal gyrus (STG), which acts as a hub for auditory-motor integration; the supramarginal gyrus in phonological short-term memory, and the opercular part of the IFG for articulatory planning [31,32]. In developmental dyslexia, brain hypoactivation, even when the task involves spoken words (auditory processing), is often observed in the left ventral occipitotemporal area, inferior temporal, and temporoparietal cortex [33]. Conversely, increased activation in the left precentral, postcentral gyri, and right STG may be a compensatory mechanism for dysfunction in the left posterior regions [34]. A core area of research focuses on atypical neural activity in regions such as the IFG associated with auditory processing, which is fundamental for accurate phonological encoding [14,35].

EEG studies investigate how oscillatory entrainment (synchronization) to speech rhythms is altered in children with DD, particularly in specific frequency bands [36,37,38]. Efficient auditory processing, which is crucial for accurate encoding of the phonological structure of words, is systematically atypical in children with dyslexia [39]. EEG research has shown that the brain’s oscillatory entrainment (synchronization) to speech rhythms is often altered [36,37,38]. Listeners rely on cortical oscillations in the delta (1–4 Hz) and low-frequency gamma (>30 Hz) ranges to process syllables (around 4 Hz) and phonemes (around 35 Hz) [37,40]. The auditory sampling in the delta/theta range may be altered, specifically affecting syllabic sampling at the delta frequency rate [35,37]. In the low gamma range, the phonemic sampling may be too slow (reducing discrimination) or too fast (overloading the auditory system) compared to controls [35,36]. These atypical neural mechanisms reinforce the idea that the phonological deficits in dyslexia are not necessarily a direct result of disturbed magnocellular function alone [35,41]. Identifying these neural differences between dyslexic types is crucial for a better understanding of the neurobiological basis of the disorder [12]. Changes in the speed of auditory sampling—such as slower phonemic sampling (reduced discrimination) or too rapid sampling (overloading the system)—are hypothesized to directly impact phonological processing in dyslexia [35,36,37]. While these local activation patterns provide a map of regional dysfunction, the deficits in dyslexia are increasingly viewed as a poor communication or (“a disconnection syndrome” [42]). Therefore, it is essential to examine the global functional connectome—the complex network of distributed regions working in concert.

### 1.3. The Functional Connectome in Dyslexia

Modern neural models of language processing posit a complex network involving distributed cortical regions, including the IFG, superior temporal gyrus (STG), and temporoparietal areas, which are typically highly integrated [27,31,32]. To characterize the efficiency of these neural pathways, brain activity is increasingly modeled as a graph consisting of nodes (representing brain regions) and edges (representing functional connections [43,44]). In this framework, Betweenness Centrality (BC) identifies ‘hubs’—nodes that act as critical bridges for information flow. Characteristic path length measures global integration, where shorter paths indicate more efficient communication, while clustering coefficients reflect functional segregation or local processing specialization [43,44].

Because it does not require active attention to stimuli, the resting-state network (RSN) is particularly well-suited for studying the emerging language system in young children [45]. Using this method, researchers have identified bilateral temporal auditory networks as early as the toddler years [46], with specific early-life experiences further shaping the maturation of these networks [45]. Compared to a state of rest, the processing of speech typically activates a distributed network of frontotemporal and parietal language regions. This system exhibits an initial right-lateralization in early childhood that progressively shifts toward left-lateralization during later development [45]. Throughout typical development, these brain networks undergo a “fine-tuning” process, transitioning from a reliance on local, proximity-based connections to the establishment of long-range, functionally specialized pathways that characterize an efficient adult-like reading system [42]. However, individuals with DD represent a deviation from this typical increase in network efficiency. DD often shows reduced connectivity in the “Reading Network” even when not reading, suggesting a structural or intrinsic vulnerability [45].

Research leveraging functional connectivity (FC) has demonstrated that individuals with DD exhibit an atypical organization of the auditory processing pathways, often characterized by reduced long-range connectivity and an over-reliance on local, effortful processing pathways [47,48,49]. While neurotypical readers automate word recognition through the ventral stream, DD brains often show “fragmented” networks that require more effortful connections to frontal areas [50]. While control readers exhibit balanced, globally integrated networks that efficiently leverage both sensory and central hubs for information flow during pure-tone discrimination, individuals with DD frequently display networks that are constrained, left-lateralized, more integrated, and less functionally specialized [50]. This constrained network suggests that individuals with DD compensate for reduced automaticity by channeling critical information through effortful executive and articulatory planning regions. Despite these insights, the precise differences in the functional hub organization in beta and gamma frequency networks during a task of phonetic binding—especially for phonologically challenging pseudowords—remain not fully understood.

### 1.4. Hypotheses and Goals of the Study

This study investigates auditory speech-EEG networks during the processing of natural words and pseudo-words in Bulgarian (a shallow orthography). By analyzing how the brain’s frequency networks entrain to natural and novel speech inputs, we expect specifically the delta/theta, beta, and gamma frequency networks to reveal clear differences between dyslexic children and controls, offering insights into the underlying phonological deficits.

The goal of the current study is to investigate the differences in functional connectivity organization and functional hubs, as quantified by Betweenness Centrality (BC) ant their strength, in the δ (1.5–4 Hz), θ (4–8 Hz), β (13–30 Hz) and γ1 (30–45 Hz) frequency networks, while control children and children with DD perform auditory short word/pseudoword discrimination. We hypothesize that these differences reflect distinct communication strategies:

**Hypothesis** **1:** *The δ network in children with DD will exhibit an inefficient functional organization during word processing, characterized by excessive local clustering and an inefficiently long characteristic path length, particularly over temporal and temporoparietal regions. The inefficient functional organization will provide the topological basis for the impaired syllabic tracking (delta-entrainment) and efficient temporal integration of phonological features.*

**Hypothesis** **2:** *The θ network in children with DD will show reduced functional segregation during pseudoword processing. This failure to establish specialized local modules will be evidenced by reduced functional hub strength (BC), particularly in frontoparietal regions (e.g., IFG, IPL), reflecting an impaired verbal working memory capacity and inefficient phonological rehearsal.*

**Hypothesis** **3:** *The β network in the DD group will be significantly left-lateralized and motor-heavy across both word and pseudoword processing, with communication channeled through the core language and sensorimotor regions, in stark contrast to the diffuse, bilateral beta hub distribution observed in controls.*

**Hypothesis** **4:** *Control children will exhibit a balanced, sensory-integrative γ1 network characterized by high BC in both posterior sensory hubs (e.g., superior occipital gyrus, SOG; occipital lobe, OL) and central/temporal integration hubs (e.g., C1/C2, MTG), demonstrating efficient, distributed information flow for phonetic feature binding as having networks that achieve an optimal balance and prioritize fast global communication.*

**Hypothesis** **5:** *Children with DD will demonstrate a restricted, executive-motor-dominated γ1 network, lacking strong posterior sensory hubs and showing significant mediation limited to core speech and motor planning areas (e.g., IFG, PstCG). This reflects a high degree of reliance on compensatory articulatory strategies for phonetic (frontal) binding, which is required for both words and novel pseudowords, making it suitable for a general mechanism hypothesis.*

## 2. Materials and Methods

### 2.1. Auditory Discrimination of Monosyllabic Stimuli (Words and Pseudowords)

All participants completed an auditory discrimination task involving monosyllabic stimuli (natural words and novel pseudowords) delivered binaurally (to both ears). The stimuli were authentic recordings of a native Bulgarian child speaking in a professional studio (44 kHz sampling rate) to maintain natural vocal qualities. The stimuli consisted of 20 real Bulgarian words with semantic content and 20 corresponding pseudowords (80 trials total). Stimuli content real Bulgarian words (20 words/40 trials) with semantic content (mean duration: 0.711 ± 0.12 ms; mean modal frequency: 1.462 ± 0.22 Hz). The pseudowords were developed specifically for this study by an experienced speech-language pathologist (logopedist), who manually substituted vowels within the real words to ensure that the resulting pseudowords followed the phonotactic rules of the Bulgarian language and were perceived as acceptable, ‘pronounceable’ speech units despite lacking meaning. The pseudowords were with mean duration of 0.725 ± 0.13 ms, and a mean modal frequency of 1.417 ± 0.24 Hz. Words and pseudowords were carefully matched for fundamental acoustic features and selected to avoid overlapping with common orthographic or phonological neighbors (Supplementary Figure S1). Stimuli were presented via Sound Blaster Z SE speakers and an audio processor (cat. No. SB1500, Creative Technology Ltd., Singapore) in a soundproof booth, with sound level calibrated to 65 dB SPL using a sound level meter (Brüel & Kjaer, mod. 2238; MicroPrecision, Grass Valley, CA, USA). The experiment consisted of 80 total trials (with stimuli repeated), divided into two to four blocks. The total duration of the auditory task was kept relatively short (~3 min), with scheduled breaks (~5 min) between blocks to mitigate the impact of cognitive fatigue. Words and pseudowords were presented in a pseudo-random order. The interval between one stimulus and the next (Interstimulus Interval, ISI) was also randomized to range from 1.5 to 2.5 s. Participants performed an auditory discrimination task using two buttons: the right button for real words listening and the left button for pseudowords listening. Hand use was not strictly controlled (usually right hand for words, left hand for pseudowords), ensuring that the neural activity recorded reflects linguistic processing rather than motor actions. The analysis window (800 ms after the stimulus onset) ends well before the average motor response (~1200 ms), so the hand-stimulus mapping did not influence the functional connectivity results. The experimenter monitored the percentage of correct responses at the end of each session.

### 2.2. Stimuli Characteristics and Creation

Words were selected to be age-appropriate and represented a wide range of parts of speech (nouns, verbs, adjectives, prepositions; Table 1) based on frequency according to a Bulgarian Frequency Dictionary [51]. This creation process ensured that the pseudowords were perceived as acceptable and “pronounceable” speech units despite having no semantic meaning. This consistency across words vs. pseudowords (Supplementary Figure S1) was demonstrated by the spectral-temporal profiles (the “shape” and “timing”), which remain highly similar and confirm the stimuli as valid Bulgarian-like pseudowords. By maintaining these specific frequency profiles, it is ensured that the differences observed in participant responses were due to the linguistic status (word vs. pseudoword) rather than any audible difference in the recording quality or pitch.

The Frequency dictionary is derived from 100,000 words of conversational Bulgarian, divided into five subgroups of 20,000 words each. Each word is assigned a rank (lower rank = higher frequency of use). Word frequency is reported as a fraction, where the numerator indicates the total number of times the word appeared in the 100,000-word corpus, and the denominator indicates the number of subgroups (maximum of 5) in which the word appeared. Words were categorized into three frequency group ranges based on the dictionary data (Table 1).

### 2.3. Selection of Groups

The eligibility of all participating children for this quantitative cross-sectional study was established by administering a comprehensive neuropsychological examination [52]. These evaluations were performed by clinical psychologists and speech-language pathologists specialized in developmental disorders. The study comprised two groups. The group with DD consisted of 24 participants (15 boys and 8 girls), with a mean age of 102 (±4) months. The control group consisted of 20 normolexic children (11 boys and 9 girls), with a comparable mean age of 101 (±3) months. Both the DD and control groups consisted of second-grade students. All participants were right-handed [53], native Bulgarian speakers, had normal or corrected-to-normal vision, and had no documented neurological or psychological deficits. The instructions for the participants were to follow specific procedures to maintain data quality and minimize artifacts. They were required to fix their eyes on a blank screen in front of them throughout the task and were only permitted to blink during the Interstimulus Interval (ISI). This rigorous control of eye movements was essential for preventing ocular artifacts from corrupting the electrophysiological recordings during stimulus presentation.

### 2.4. Instruments

#### 2.4.1. Participant Groups and Initial Assessment

The group with DD was created by selecting children who displayed significant difficulties in reading. While the children had histories of literacy struggles in the school setting, their diagnosis of DD for the purposes of this study was confirmed through the standardized testing battery. Specifically, inclusion required participants to have scores below the average range (i.e., more than one standard deviation below the mean for their age) on standardized measures of both reading speed and reading accuracy. This deficiency had to be confirmed by performance on two separate assessment tools: (1) The Dyslexia & Developmental Dysorthography Assessment Battery-Second Edition (DDE-2) [54,55] (Supplementary Figure S2); (2) A “Reading Abilities” test [56] (Supplementary Figure S3).

The normolexic control group consisted of children who were matched to the DD group based on their age and socio-demographic backgrounds. Every child in the control group demonstrated typical reading performance, scoring within the normal range on both the DDE-2 and “Reading Abilities” assessments. The control group specifically excluded any children diagnosed with dyslexia or any co-occurring language disorders.

#### 2.4.2. Diagnostic and Psychometric Measures

The children in the study underwent a standardized testing battery to confirm diagnosis, assess abilities, and control for non-verbal intelligence. The diagnostic and skills were assessed by the battery administered to all children included: (1) The Dyslexia & Developmental Dysorthography Assessment Battery-Second Edition (DDE-2) [54,55], a key tool for diagnosing and characterizing developmental dyslexia; (2) The “Reading Abilities” test [55]; (3) The Girolami-Boulinier’s test for non-verbal perception with “differently oriented signs” [56,57], which assesses visual-perceptual skills. The results from this battery demonstrated a wide range of reading and writing difficulties within the Developmental Dyslexia (DD) group (Supplementary Figure S2). Non-verbal intelligence test was performed additionally for all participants, who completed Raven’s Progressive Color Matrices [58]. This assessment was used to ensuring that differences observed in the main task were not attributable to variations in general cognitive ability.

All participants underwent a psychometric test battery tailored for primary school children to assess their reading, writing, and phonological awareness [56] (Supplementary Figure S3). The battery included specific tasks to evaluate literacy skills: (1) reading fluency was assessed through the “Reading Abilities” test, which required children to read a 133-word text aloud; (2) writing ability was evaluated through a dictation task consisting of 30 sentences that required the child to supply missing compound words; (3) phonological awareness was tested through tasks that required children to manipulate word sounds, such as identifying words or omitting either the initial sound (“without the first sound-letter”) or the final syllable (“without the last syllable”) of a given word. Significant differences were found between the group with DD and the control group in both the accuracy and the speed with which they completed these psychometric tasks (Supplementary Figure S3). The children in the DD group achieved normal nonverbal intelligence quotient (IQ) scores (IQ ≥ 98, Supplementary Figure S3) on the Raven’s progressive color matrices [59]. This finding is consistent with age-appropriate norms and confirms that the observed literacy difficulties were specific to reading and language processing, not due to a general cognitive deficit.

### 2.5. EEG Data Acquisition and Processing

#### 2.5.1. EEG Data Acquisition

The electroencephalography (EEG) signals were acquired using a 40-channel wireless system [60] that features specialized star-shaped gold-dry sensors. Data were collected at a sampling rate of 250 Hz. The electrode arrangement was consistent with a combination of the standard international systems 10–10 and 10–20 International Systems [61,62]. This arrangement spanned brain locations and included 10/10 sites: AF3–AF4, F7–F8, C1–C2, C5–C6, P7–P8, PO3–PO4; 10/20 sites: Fz, F3–F4, Cz, T7–T8, Pz, O1–O2. The reference electrodes were positioned on the processus mastoidei, while the ground electrode was placed on the forehead. To effectively eliminate the issue of volume conduction, online re-referencing to a global median reference was applied [63]. To maintain high signal quality throughout the recording, skin impedance was strictly kept below 5 kΩ.

#### 2.5.2. EEG Data Preprocessing

The EEG signals underwent several preprocessing steps to prepare the data for analysis and ensure high quality. Individual trials were segmented into 800 ms epochs, which were synchronized to the onset of the auditory stimulus. Functional connectivity analysis was performed on this interval to encompass the essential stages of speech perception and lexical discrimination. This provided a conservative time window prior to the average behavioral reaction time (mean RT ≈ 1200 ms), effectively isolating cognitive-linguistic networks from motor-related activity. The data were band-pass filtered (1–70 Hz, using a Chebyshev II filter; MATLAB R2023b, The MathWorks, Inc., Natick, MA, USA), and a 50 Hz notch filter was applied to remove electrical line noise. To analyze specific brain rhythms, the data were then separated into seven distinct frequency bands. This was achieved by using a 4th-order Butterworth filter (MATLAB R2023b, The MathWorks, Inc., Natick, MA, USA), which was applied in both the forward and backward directions to prevent artificial phase shifts. The analyzed bands were δ (1.5–4 Hz), θ (4–8 Hz), α (8–13 Hz), β1 (13–20 Hz), β2 (20–30 Hz), γ1 (30–48 Hz), and γ2 (52–70 Hz). We acknowledge that the connectivity patterns for the beta and gamma sub-bands exhibit similar trends in our current findings. The subdivisions (β1/β2 and γ1/γ2) are driven by distinct neural generators and serve different functional roles. Specifically, in language tasks, β1 is often related to the stabilization of mental representations, whereas β2 is frequently associated with higher-order cognitive demands and the processing of novel or unexpected information. Similarly, γ1 is more closely linked to lexical-semantic integration, while γ2 reflects local acoustic-phonetic processing. Maintaining these subdivisions ensures that subtle, frequency-specific effects relevant to the neurobiology of developmental dyslexia are not masked.

Strict criteria were applied to remove contaminated data and preserve only quality, task-relevant trials: (1) By amplitude threshold, trials containing artifacts (like eye blinks or muscle activity) with amplitudes exceeding ±200 µV were rejected; (2) Each trial also had to meet a specific signal-to-noise ratio (SNR) criterion [64]. The noise for each sensor was determined by calculating the standard deviation of the residuals remaining after subtracting the average signal across all trials from the individual trial signal. The SNR was then calculated by dividing the peak-to-peak amplitude of the mean signal by twice the standard deviation of the noise. Only trials that passed all noise removal criteria and corresponded to a correct behavioral response were retained for subsequent analysis.

#### 2.5.3. Analyzed Data and Frequency Bands

Following the artifact rejection and preprocessing steps, the number of clean (artifact-free) data epochs available for analysis differed significantly between the groups: For controls, the number of epochs per condition and subject was 32 ± 5 (mean ± standard deviation). For children with DD, the number of epochs per condition and subject was 21 ± 7. To compensate for this imbalance and ensure statistical comparability, additional recording sessions were conducted specifically with the children in the DD group until the number of clean epochs was roughly equal across both groups. The balanced EEG data were then analyzed within the seven frequency bands δ, θ, α, β1, β2, γ1, and γ2 Hz.

### 2.6. Functional Connectivity

Functional connectivity between the recorded brain regions was assessed using the Weighted Phase Lag Index (wPLI) [65,66]. The wPLI is a robust measure designed to quantify the extent of phase synchronization between the EEG signals of two different sensors. By focusing on the asymmetry of the phase distribution and ignoring zero-lag correlations, the wPLI inherently effectively reduces the bias introduced by volume conduction and signal leakage, which are common artifacts in sensor-space EEG recordings [65,66]. The wPLI was computed as a fraction, with the numerator capturing the magnitude of phase lag asymmetry and the denominator scaling the result to ensure the wPLI value falls within the range of 0 to 1. A value near 0 suggests an absence of a consistent phase relationship, while a value closer to 1 indicates strong and consistent phase synchronization between the two brain regions. A separate adjacency matrix was constructed for every possible pair of EEG sensors, for each time series, and for each frequency band. The resulting wPLI values formed the entries of the weighted adjacency matrix. This matrix, which represents the strength of connectivity between all electrode pairs, was then used as the basis for calculating the network’s Small-World Propensity (SWP) [67].

The Small-World Propensity (SWP, ϕ) [67] was used to quantify the efficiency of information transfer within the brain’s functional network. A network exhibits small-world characteristics when it successfully balances two competing properties. A high clustering coefficient signifies dense local connections, enabling specialized local information processing. Short characteristic path length indicates efficient global communication across the network (fast information transfer between distant nodes). This optimal balance allows the brain to perform both specialized local processing and rapid, integrated global communication [67]. SWP quantitatively assesses how closely an observed network adheres to this ideal small-world balance. SWP was calculated by evaluating the deviation of the observed network clustering coefficient (∆C) and characteristic path length (∆L) from theoretical random (Crand, Lrand) and regular (Clatt, Llatt) network models. High SWP (ϕ ≈ 1): A high value indicates robust small-world attributes, which means the network has a high observed clustering coefficient (Cobs) combined with a short to moderate observed path length (Lobs), representing an efficient and balanced network. The low SWP (ϕ ≈ 0) signified a weaker small-world propensity [67], which corresponded to large deviations (∆L, ∆C) from the null models, suggesting a less optimal balance between local segregation and global integration. By normalizing these metrics against 100 randomized null-model networks with identical degree distributions, the non-random topological features were isolated. This process ensured that group differences in functional segregation (∆C) and global integration (∆L) were not artifacts of network density, but represented true differences in neural specialization and communication efficiency [44,67].

For every subject, the global connectivity characteristics were extracted from the single-trial wPLI matrices for each of the frequency-specific networks. These metrics were derived using specific MATLAB R2023b (The MathWorks, Inc., Natick, MA, USA) functions available in the Brain Connectivity Toolbox (version 2019-03-03 [67]). The global measures were statistically compared between the study groups using nonparametric methods.

Local node metrics were used to identify the most critical nodes (brain regions) and edges (connections) within the functional networks. Two local measures were calculated for each electrode (node). The strength of the node was defined as the sum of weights (wPLI values) of all connections leading to or from that node. The Betweenness Centrality (BC) quantifies the importance of a node in network communication. It is the proportion of all shortest paths in the entire network that pass directly through that specific node [43,44,68]. Both strength and BC values were calculated using the weights in the adjacency matrix and then normalized for each subject (by dividing the node’s score by the average score across all nodes). Nodes with both high strength and high BC are critical for information processing, as they participate in a large number of the shortest paths [69]. Hubs were established as nodes whose average strength or BC (calculated across all participants) was at least one standard deviation above the overall group average for that specific measure [44]. A higher maximum BC or strength is generally indicative of greater network integration [43,68]. The most important links (edges) were defined as connections whose BC, averaged across all participants, was at least one standard deviation above the mean group edge BC.

The final characteristics of the individual brain networks (for each participant, frequency band, and auditory stimulus) were established by integrating anatomical parcellation methods, brain mapping techniques, and the calculated connectivity measures (wPLI, BC, and strength). All these properties were processed and summarized using the BrainNet Viewer functional package 1.7 [69] to map the functional networks onto a standardized brain template. The characteristics (strength, BC) of the nodes (representing brain regions/sensors) were indicated by their size and color. Edges (representing functional connections) were also mapped, with their properties (strength) represented by their size and color.

### 2.7. Statistical Analysis

During EEG recording sessions, Kruskal–Wallis non-parametric tests (KW test) were used to compare reaction times and performance accuracy between control and DD groups for each condition (2) [70]. Subsequently, a nonparametric procedure with 1000 permutations was employed for between-group pairwise comparisons of each global SWP measure (ϕ, ∆L, ∆C) across each frequency band and word/pseudoword condition [71,72]. The Bonferroni correction was applied to control the family-wise error rate (FWER) in multiple hypothesis testing. The adjusted significance level was calculated as *p* = α/3 = 0.05/3 ≈ 0.0167 to account for the three dependent measures. Furthermore, Benjamini and Hochberg’s procedure [73] was also used to control the false discovery rate (FDR) within a family of hypothesis tests.

Local measures of individual nodes were assessed using statistical methods based on non-parametric permutation analyses with cluster-mass tests [70,72]. Hubs were identified as nodes where the maximum strength or BC value was at least one standard deviation greater than their respective group average for that measure. Significant clusters were identified by applying a critical value to the maximum cluster statistic, with the false alarm rate controlled through multiple comparison corrections. The nodes within these clusters were indexed according to their corresponding EEG sensors, and the medians of their distributions were used to examine hemispheric differences. Permutation tests with cluster-mass tests were employed to effectively control the FWER and address the multiple comparisons problem for the dependent local measures (adjusted significance level: *p* = α/2 = 0.025). An additional procedure [73] was used to control the FDR within a family of hypothesis tests. The same selective criteria were applied to the links and edges presented in the figures, highlighting those with specific values of BC and strength (Str).

## 3. Results

### 3.1. Behavior Parameters in Auditory Discrimination of Words and Pseudowords

Children with DD were significantly faster than controls for both word listening (mean ± s.e., DD: 1072.01 ± 26.36 ms; controls: 1253.85 ± 17.17 ms; *χ*^2^ = 25.94; *p* = 3.51 × 10^−7^), and pseudoword listening (DD: 1097.56 ± 30.59 ms; controls: 1245.43 ± 35.47 ms; *χ*^2^ = 31.1; *p* = 1.1 × 10^−8^). The control group demonstrated significantly higher discrimination accuracy compared to the children with DD for word (children with DD, 61.88 ± 3.65%; controls, 81.8 ± 1.58%;, *χ*^2^ = 19.92; *p* = 8.1 × 10^−6^), and pseudoword listening (DD: 60.31 ± 4.71%; controls: 78.4 ± 2.39%; *χ*^2^ = 7.64; *p* = 0.005).

The DD group displayed faster reaction times but significantly lower accuracy. This “impulsive” behavioral pattern suggests a shallower processing strategy or non-lexical guessing, wherein children with DD may sacrifice accuracy for speed during rapid auditory or visual discrimination tasks [74,75,76]. To account for this speed-accuracy trade-off, behavioral efficiency was quantified using the Inverse Efficiency Score (IES), defined as the ratio of mean reaction time to the proportion of correct responses [75]. For word listening, the DD group showed a lower IES (995.77 ± 3.67 ms, which accounted for 64.4% of the variance in the regression model (adjusted R^2^ = 0.644), compared to controls (1123.1 ± 7.08 ms, adjR^2^ = 0.679; Supplementary Figures S4–S6, Tables S1–S4). For the more demanding pseudoword condition, the explanatory power of the model for the DD group dropped to 56.4% (IES: 1035.3 ± 3.47 ms), while it remained high for controls at 70% (IES: 997.47 ± 3.84 ms). Across all groups and conditions, IES served as a predictor in the regression models (R^2^ ranging from 0.57 to 0.71). The negative slopes (ranging from −1632.6 to −2452.9) indicate that as efficiency improves (indicated by lower IESs), neural performance measures increase significantly (*p* < 0.001). This confirmed that IES effectively captures the underlying processing capacity of both groups across word and pseudoword conditions (Supplementary Figures S4–S6, Tables S1–S4).

The IES suggested that the DD group’s efficiency is highly sensitive to task complexity. The decrease in the model’s explanatory power for the DD group during pseudoword listening (adjR^2^ dropping from 64.4% to 56.4%) was consistent with the hypothesis of a less robust linguistic memory system [74,75,76].

### 3.2. Global Measures During Auditory Discrimination of Monosyllabic Words

**Figure 1:**
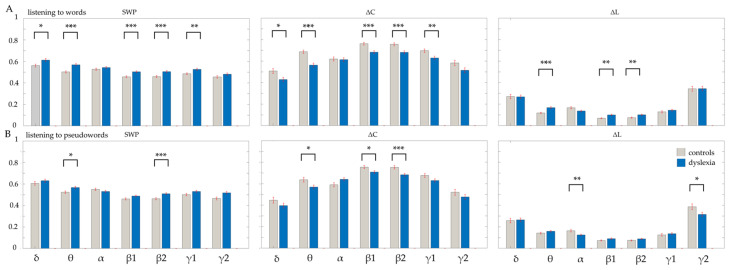
Comparison of Global Network Topology across Frequency Bands. Differences in global network organization across the frequency spectrum (δ, θ, α, β1, β2, γ1, and γ2). Data represent Controls (gray bars) versus children with DD (blue bars) for: (**A**) Auditory discrimination of monosyllabic words; (**B**) Auditory discrimination of pseudowords. Metrics: SWP (Small-World Propensity), ∆C (deviation of network’s clustering coefficient); ∆L (deviation of network’s characteristic path length). Data are shown as means ± s.e. Significant differences are indicated by asterisks (* *p* < 0.05, ** *p* < 0.01, *** *p* < 0.001).

For the children with DD, a significantly higher ϕ was observed in the δ-frequency and γ1-frequency networks compared to the control group. This higher ϕ was driven by significantly reduced functional segregation (smaller ΔC) in the DD network relative to Controls (δ- and γ1-networks: ϕ and ΔC, *χ*^2^ > 6.4295; *p* < 0.0112; Table 2; Figure 1A). The DD network exhibited lower local connectivity and clustering compared to the Control network.

For the θ- and β-frequency networks of the children with DD, we observed a higher ϕ alongside a higher ΔL, and a smaller ΔC compared to the control children (θ– and β-networks: ϕ, ΔC, and ΔL, *χ*^2^ > 10.573; *p* < 0.0011; Table 2; Figure 1A). The higher ϕ was achieved through a unique trade-off, characterized by significantly reduced functional segregation (lower ΔC) and reduced functional integration (higher ΔL) compared to the control network.

### 3.3. Global Measures During Auditory Discrimination of Monosyllabic Pseudowords

In the θ and β2- frequency networks, the controls displayed lower ϕ and higher ΔC than the children with DD; both groups had similar ΔL. The controls exhibited a more efficiently segregated network, which means that their local brain regions are more specialized and closely clustered, which, characterizing efficient brain functions (θ- and β2-networks: ϕ and ΔC, *χ*^2^ > 5.7753; *p* < 0.0162; Table 3; Figure 1B). The children with DD showed a less specialized local network (smaller ΔC), suggesting less efficient processing within functional brain modules. The networks of the DD group demonstrated a clear deficit in functional segregation, while global communication (ΔL) being statistically similar in both groups (*χ*^2^ < 4.751; *p* > 0.0292). This pattern specifically highlights a deficit in local efficiency in the networks of children with DD.

The β1-frequency network in control subjects exhibited a higher ΔC compared to children with developmental dyslexia (DD) (*χ*^2^ = 6.1522; *p* = 0.0131; Table 3; Figure 1B). Both groups showed similar ΔL and ϕ values (*χ*^2^ < 5.2285; *p* > 0.0222). The key difference in the β1 frequency band was the reduced functional segregation (lower ΔC) in children with DD. This finding suggests a challenge in establishing specialized, efficient local modules that are essential for specific cognitive tasks. Overall, the brain networks in children with DD demonstrate less efficient organization at the local level compared to those of typical readers, although long-distance connectivity appears to be normal.

In the γ2-frequency network, the controls showed significantly higher ΔL than children with DD (*χ*^2^ = 6.0045; *p* = 0.0142; Table 3; Figure 1B). However, both groups had statistically similar ΔC and ϕ values (*χ*^2^ < 5.566; *p* > 0.0183). The result in the γ2 band suggests that local processing (ΔC) is maintained in children with DD compared to controls. The difference lies in global integration (ΔL). The control network used more complex routing for information processing in this high-frequency band. The network of children with DD used a shorter, simpler, or less mature routing strategy, which artificially lowered ΔL.

### 3.4. Assessing the Predictive Relationship Between Small-World Propensity and Behavioral Efficiency

To investigate the neural efficiency of auditory processing, we linked the SWP connectivity metric to behavioral IES using a series of robust regression models [75]. Robust regression modeling revealed a critical “tipping point” in neural efficiency during word listening for the control group (Supplementary Figures S4 and S5; Table S1). For all frequency bands up to β1, increased SWP (ϕ) was related to enhanced efficiency (lower IES). However, in higher frequencies, high ϕ predicted lower efficiency (higher IES) for word listening. The results indicated that during auditory discrimination of pseudowords, high SWP in beta bands led to lower efficiency (higher IES), while in all other frequency bands, higher SWP led to lower IES, indicating higher processing efficiency (Supplementary Figures S4–S6; Table S2).

The group with DD exhibited that the high SWP in the delta band was strongly associated with lower efficiency (higher IES) across both conditions (words: *p* = 0.002, Supplementary Figure S5, Table S3; pseudowords: *p* < 0.001; Supplementary Figure S6, Table S4). This “efficiency reversal” suggested that while a small-world topology is generally considered optimal, in the dyslexic brain, increased low-frequency synchronization may represent a compensatory but inefficient strategy, or perhaps a “neuronal noisy” that hinders rapid phonological processing. During word listening, the group with DD showed a significant positive slope for β2 (slope = +2089.1, *p* < 0.001, Supplementary Figure S5, Table S3). Like the controls at high frequencies, the brain of children with DD became less efficient (higher IES) in the β2 band at processing real words. During pseudoword listening, the relationship to β2 in the dyslexia actually reversed (slope = −1874.2, *p* < 0.001; Supplementary Figure S6, Table S4). While the association with β1 reflected diminished efficiency (higher IES), suggesting a decoupling of neural activity from optimal behavioral outcomes. For non-lexical stimuli (pseudowords), a small-world organization in the β2 band suddenly became beneficial (lower IES).

### 3.5. Local Measures of the Frequency Functional EEG Networks During Auditory Word Discrimination

**Figure 2:**
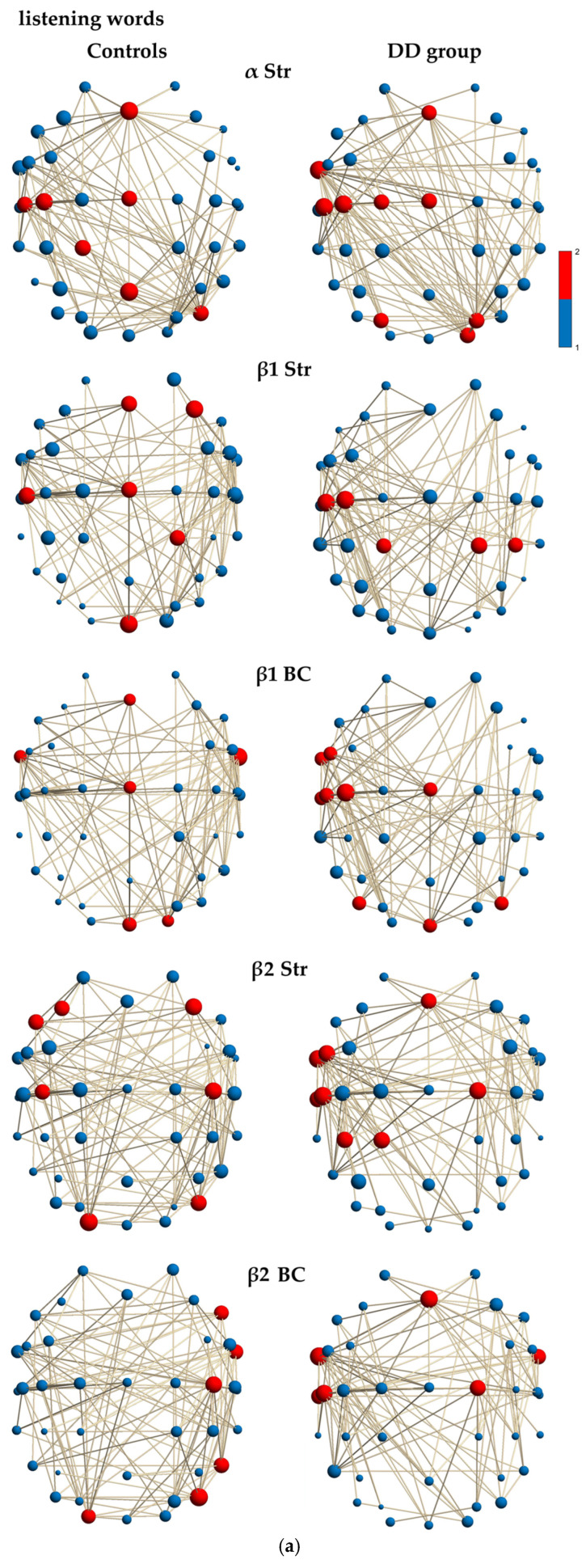

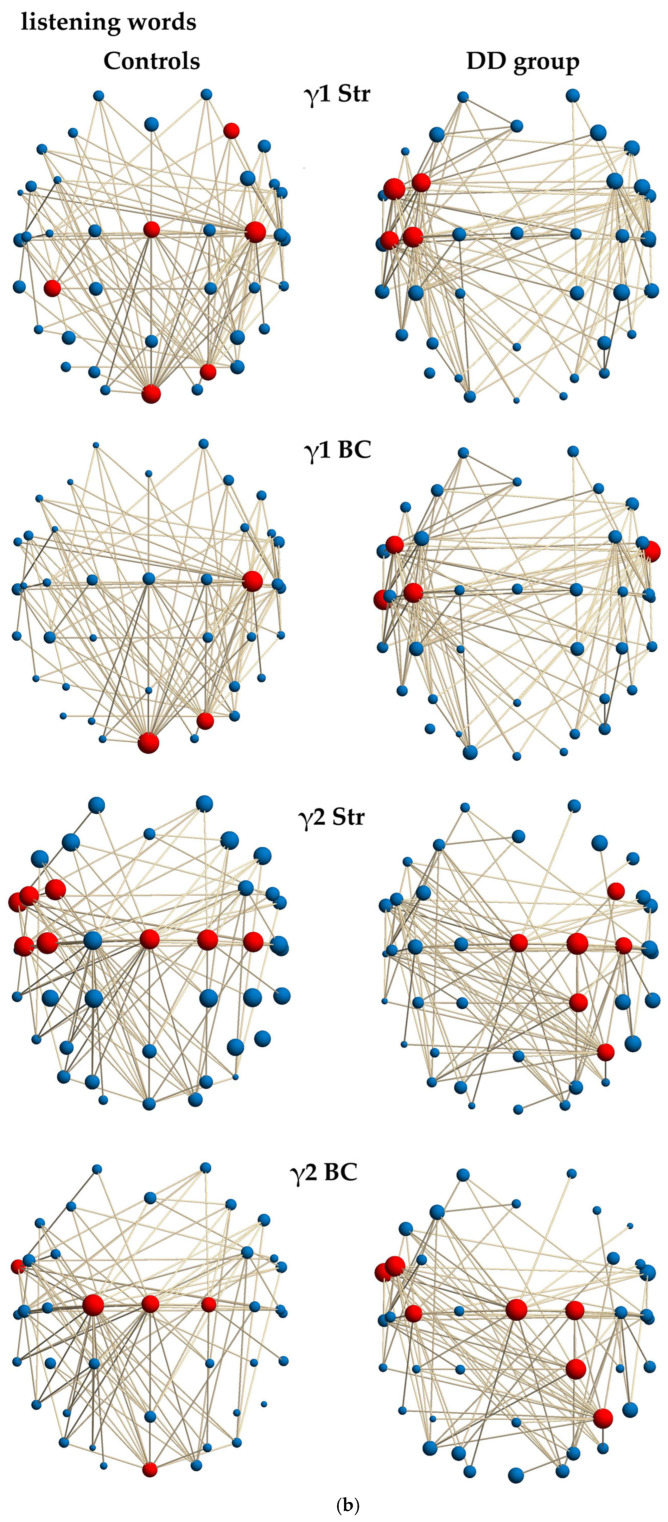
(**a**) Low frequency networks of controls (1st column) and children with DD (2nd column) during listening of monosyllabic words. The 3D spatial network topology of nodes is shown in scalp coordinates, using a color gradient from red to blue. Red nodes represent main hubs and black connections- strong connections that are above the significant threshold; their largest size corresponds to a value of 2 on the color scale. Blue nodes and gray links are below the significant threshold, with the smallest size representing a value of 1: (1) α-network: Str of the hubs: Controls: Fz, Cz, C3, C5, CP1, Pz, P08; Children with DD: FT9, Fz, C1, Cz, C3, C5, P03–04, O2. (2) β1-network: Str of the hubs: Controls: Fz, F4, Cz, C5, CP2, Oz; Children with DD: C3, C5, CP1–2, CP4. (3) BC of the hubs: Controls: Fz, FT9–10, Cz, Oz, O2; Children with DD: FC5, FT9, Cz, C3, C5, T7, P07–P08, Oz. (4) β2-network: Str of the hubs: Controls: F3–4, F7, C3–4, P08, O1; Children with DD: Fz, FC5, FT9, C2, C5, CP1, CP3, T7. (5) BC of the hubs: Controls: F8, FT10, C4, P08, P8, O1; Children with DD: Fz, FT9–10, C5, C2, T7. (**b**) High frequency networks in controls and children with DD during listening of monosyllabic words: (1) γ1-network: Str of hubs: Controls: F4, Cz, C4, CP3, P04, Oz. Children with DD: FC3, FC5, C3, C5; (2) BC of hubs: Controls: C4, P04, Oz. Children with DD: FC5, FT10, C3, T7; (3) γ2-network: Str of hubs: Controls: FC3, FC5, FT9, C2, Cz, C3–C4, C5; Children with DD: FC4, C2, Cz, C4, CP2, P4; (4) BC of hubs: Controls: FT9, C1, Cz, Oz, C2; Children with DD: FC5, FT9, C2, C3, Cz, CP2, P4.

The local characteristics of the frequency-functional EEG networks were analyzed using the Kruskal–Wallis non-parametric test to compare the median distributions of key brain areas (hubs) between controls and children with DD (Table 4). This allowed us to specifically describe the differences in hub topology within the α-, β- and γ-frequency networks involved in the auditory discrimination of monosyllabic words (Figure 2a,b).

#### 3.5.1. Alpha-Frequency Network During Auditory Word Discrimination

In the α-frequency network (Str; *χ*^2^ = 6.114; *p* = 0.0134; Table 4; Figure 2a), the analysis revealed statistically significant differences in hub topology between the groups. The control group exhibited strength hubs that were widely distributed across central and midline regions, indicating a balanced and efficient network architecture. Key hubs (Fz, Cz, and Pz) effectively connected regions related to the executive function (Medial Frontal Cortex, MFC: Fz), sensorimotor processing (Postcentral Gyrus, PstCG: Cz, C3, C5), attention and spatial mapping (Superior Parietal Lobe, SPL: CP1, Pz), and the right Middle Occipital Gyrus (MOG: PO8). This topology reflected optimal coordination of resources that supported the left-lateralized phonological loop (with PstCG/SPL involvement for rehearsal and somatosensory integration) and the temporal coordination of attention necessary for complex auditory analysis.

In contrast, the DD group’s hubs in the α-frequency network displayed a significantly different, more heterogeneous distribution, suggesting an atypical network organization. This was characterized by a strong and unique involvement of the left anterior part of the Inferior Temporal Gyrus (aITG: FT9), which functioned as an atypical hub for language and auditory processing. There was an increased emphasis on the fronto-sensorimotor cortex, including MFC (Fz), the Precentral Gyrus (PreCG: C1), alongside the PstCG (Cz, C3, C5). This suggested a heavy and potentially effortful reliance on the articulatory system (inner speech/motor rehearsal) to compensate for underlying phonological analysis difficulties. The involvement of the Superior Occipital Gyrus (SOG: PO3-PO4) and the right Visual Cortex (OL: O2) indicated an atypical sensory focus on early sensory areas, likely serving as a compensatory mechanism for primary auditory and phonological deficits.

#### 3.5.2. Beta1-Frequency Network During Auditory Word Discrimination

The β1 networks (Str; *χ*^2^ = 21.01; *p* = 4.57 × 10^−6^; Table 4; Figure 2a) showed statistically significant differences in hub topology between the groups. The control children showed robust frontal control and a globally distributed network that supported the maintenance of phonological and attentional states. Key hubs in the medial frontal cortex (MFC: Fz, F4) exhibited higher strength, reflecting the necessary attentional and executive functions to sustain focus and analyze during word listening. The involvement of the sensorimotor cortex (Cz, C5) indicated optimal neural activity for maintaining the phonological code (inner rehearsal) and integrating it with sensory input. Hubs in the right SPL (CP2) and occipital midline (Oz) ensured a stable perceptual environment during word listening.

In contrast, the β1 network (Str) in individuals with DD revealed a strong yet isolated focus on left sensorimotor hubs (C3, C5), suggesting an over-emphasis on motor articulation planning to hold and process the sound structure of words. This finding is often interpreted as a compensatory strategy for impaired phonological decoding. The high bilateral engagement in the SPL (CP1–2) and right inferior parietal lobe (IPL, CP4) indicated that the β1 network relies on somatosensory feedback or atypical spatial attention mechanisms to manage the auditory aspects of words, deviating from the more frontal-midline dominance seen in control children. The β1 network in the group with DD was constrained and lacked the MFC(Fz) and visual input hub (Oz) seen in controls. A less integrated, localized, and compensatory strategy maintained the auditory structure of words, which was heavily dependent on physical or motor aspects of speech (PstCG: C3, C5) rather than efficient, controlled cognitive or linguistic maintenance.

The β1 network (BC; *χ*^2^ = 10.192; *p* = 0.0014, Table 4; Figure 2a) also showed statistically significant differences in hub topology between the groups. In control children, the MFC (Fz) was also seen as a primary hub for executive control, serving as a gateway for top-down attention and working memory that influences the entire network. The bilateral aITG hubs acted as a linguistic bridge, directing auditory and phonological information to frontal areas. The central PstCG hub (Cz) facilitated sensorimotor control and inner speech planning, integrating these processes with the frontal executive system. The occipital midline (Oz) and right occipital (O2) cortex were also involved in a listening task. These hubs may integrate a broad allocation of resources earlier.

In the group with DD, the β1 network (BC) displayed a left-lateralized focus on articulatory and motor regions, as well as temporal areas. The high BC in the β1 network of the left inferior frontal gyrus (IFG, FC5) indicated that information flow was heavily routed through this area, likely for effortful phonological encoding and rehearsal (inner speech). The left aITG (FT9) and middle temporal gyrus (MTG: T7) were essential hubs in the left hemisphere responsible for decoding and processing the auditory-linguistic features of words. The extensive involvement of the central and left PstCG (Cz, C3, C5) suggested that the brain was relying heavily on the motor system to “articulate” and hold words (motor compensation) due to less efficient phonological storage. Hubs in the bilateral MOG (PO7-PO8) and occipital midline (Oz) facilitated the integration of auditory, spatial, and early sensory information, linking these sensory inputs to the central language system. For children with DD, the β1 network was left-lateralized and motor-oriented, with key mediation confined to the IFG (FC5), aITG (FT9), and PstCG (C3, C5). The absence of right aITG (FT10) and MFC (Fz) hubs found in controls suggested a less integrated executive control and an impaired ability to distribute information bilaterally.

#### 3.5.3. Beta2-Frequency Network During Auditory Word Discrimination

The β2-networks between the groups were statistically different (Str; *χ*^2^ = 25.678; *p* = 4.03 × 10^−7^; Table 4; Figure 2a). Bilaterally distributed hubs across frontal (MFG: F3–F4; IFC: F7) and posterior regions, PstCG (C3–C4), (MOG: PO8; OL: O1) in the control group suggested an efficient and balanced recruitment of resources required for high-level working memory and sustained attention.

The β2 network hubs (Str) in children with DD were in the IFG (FC5), PstCG (C5), and aITG (FT9)/MTG (T7) areas, indicating that processing the heard word demanded significantly greater effort and resources in the language and motor systems for maintenance, reflecting the neural cost of overcoming phonological deficits. The lack of strong bilateral frontal hubs MFG (F3/F4) suggested less efficient and less balanced cognitive control compared to the control group. The MFG (Fz) showed centralized executive engagement, but the left IFG (FC5) indicated an intense focus on the motor planning and rehearsal component of working memory, likely reflecting greater effort. The strength of left-hemispheric language regions aITG (FT9) and posterior STG (pSTG: T7) suggested they were heavily engaged or overloaded to analyze and maintain the auditory word’s phonological structure. The hubs in the PstCG (C2, C5)/left parietal (SPL: CP1; IPL: CP3) suggested an expanded and intense dependence on the articulatory and somatosensory feedback systems to encode and sustain the phonological information in working memory.

The β2 network (BC; *χ*^2^ = 12.994; *p* = 0003; Table 4; Figure 2a) was statistically different between the groups. The control group relied on bilateral, posterior, and right-sided hubs, indicating efficient communication routing that integrates sensory input with frontal control. The β2 network (BC) of controls showed a diffuse, bilateral hub distribution with a notable right-hemispheric representation. This included language regions (IFG: F8; aITG: FT10), and alongside sensory areas (PstCG: C4; ITG: P8; MOG: PO8). The high cognitive demands were managed through a broad, highly efficient system where right-sided executive and sensory areas were critical for routing information.

The hubs in the β2 network (BC) of children with DD were strongly left-lateralized and concentrated in the frontal, temporal, and sensorimotor areas, highlighting a constrained and focused communication strategy. The MFC (Fz) served as a major frontal gateway for attention and control signals, potentially compensating for the lack of bilateral frontal hubs seen in controls. The bilateral aITG (FT9–10) with high BC here suggested these were the critical points for routing phonological information. The left aITG (FT9) indicated a highly dependent hub for auditory-linguistic input. The PreCG (C2) and PstCG (C5) hubs reflected a high reliance on the articulatory/inner speech system for information flow, a compensatory mechanism for inefficient phonological processing. The left MTG (T7, Wernicke’s area) was a central hub in the left-side language comprehension network. Its high BC indicated that all information flow associated with auditory word decoding must be routed through this critical temporal area. The β2 network of children with DD had left-lateralized hub distribution (aITG (FT9), PstCG (C5), MTG (T7)) focused on the language and articulatory circuits, along with a singular reliance on the MFC (Fz) midline hub. The network lacked the diverse, posterior hubs seen in controls, suggesting a less globally integrated communication that concentrated routing demands on a few core language/motor processing hubs.

#### 3.5.4. Gamma1-Frequency Network During Auditory Word Discrimination

The distribution of γ1 strength hubs in the control group was significantly different from that of children with developmental disabilities (DD) (Str; *χ*^2^ = 13.352; *p* = 0.003; Table 4; Figure 2b). The γ1 network in the control group displayed a widespread hub distribution that facilitated the rapid binding of phonological features with attentional control, particularly in the middle frontal gyrus (MFG: F4/PstCG: Cz, C4) and the integration of semantic-spatial context (IPL: CP3/SOG: PO4), midline occipital lobe (Oz)). The γ1 network for controls (Str) was balanced and broadly engaged, utilizing strong hubs within both the right executive medial frontal cortex (MFC: F4) and posterior sensory regions (CP3, PO4, Oz). This configuration reflected an optimal network for fast and efficient feature binding and sensory integration, which is essential for rapid word recognition.

In contrast, the γ1 hubs (Str) in children with DD were highly concentrated in the left fronto-central regions and completely excluded posterior (parietal/occipital) areas. This suggests a localized, effortful, and possibly compensatory binding mechanism that focused on motor and frontal language areas. The left IFG (FC5) and MFG (FC3) indicated a significant effort concentrated in the left frontal region, with hubs close to Broca’s area demonstrating extremely high, localized engagement of the executive/motor planning system. The left PstCG (C3, C5) revealed intense recruitment of the left motor system. The γ1 network of children with DD was highly left-lateralized and dominated by frontal and motor areas, with a complete absence of posterior (sensory) hubs, reflecting a breakdown in the efficient sensory feature binding mechanism. Instead, the network relied heavily on a constrained fronto-articulatory loop to manually bind the phonetic features of words.

The γ1 frequency network (BC) also showed significant differences between the groups (*χ*^2^ = 9.768; *p* = 0.017; Table 4; Figure 2b). In the control group, the γ1 frequency network utilized posterior (sensory) and right-central regions as key information hubs. The right PstCG (C4) facilitated both motor inhibition and global sensorimotor integration, indicating effective communication between the motor system and cognitive control. The right SOG (PO4) served as a crucial point for integrating spatial and acoustic-perceptual features. The occipital midline area (Oz) supported the efficient integration of early sensory and attentional processing with the language network, reflecting a globally connected system. The γ1 network for controls (BC) featured posterior and right-lateralized hubs, including PstCG (C4), SOG (PO4), and OL (Oz).

Conversely, The hub distribution in the γ1 network (BC) for the group with DD was predominantly left-lateralized at IFG (FC5), PstCG (C3), and MTG (T7), with communication heavily routed through the articulation and language processing areas. The left PstCG (C3) reinforced the role of the IFG (FC5), signifying that the motor system is a crucial communication pathway for binding features, often interpreted as a compensatory mechanism for weaker auditory processing. The left middle temporal gyrus (MTG: T7; Wernicke’s Area) was essential for decoding the linguistic and semantic features of words. The right aITG (FT10) indicated an atypical reliance on temporal areas for processing and relaying auditory signals. The absence of strong posterior sensory hubs (SOG: PO4; and OL: Oz) found in controls indicated a less efficient and more effortful somatomotor binding strategy that requires greater conscious effort and localized processing to group the phonetic elements of words.

#### 3.5.5. Gamma2-Frequency Network During Auditory Word Discrimination

The distribution of the γ2 strength hubs showed significant differences between the groups (Str; *χ*^2^ = 43.463; *p* = 4.3 × 10^−11^; Table 4; Figure 2b). In the control group, these hubs were highly concentrated in the left MFG (FC3), IFG (FC5), and aITG (FT9) regions. This hub distribution suggests that the fastest and most robust processing occurred within key language and articulatory circuits for decoding the heard words. The PreCG (C2) and bilateral PstCG (Cz, C3–C4, C5) demonstrated strong yet balanced recruitment of the sensorimotor areas. This pattern reflects highly efficient and automatic processing, where cognitive effort was maximally concentrated in neural circuits responsible for rapidly analyzing and coding auditory input.

The dyslexic group’s γ2 hubs (Str) exhibited a shift toward the right hemisphere and posterior (parietal) regions, with less focus on the core left language areas compared to the controls. The right hemispheric MFG (FC4), PreCG (C2), PstCG (Cz, C4), SPL (CP2), and IPL (P4) suggest that high cognitive demands were being met by atypically engaging right-sided executive, spatial, and attentional systems as a compensatory strategy for less efficient processing in the left hemisphere. The absence of strong left language hubs in the MFG (FC3), IFG (FC5), and aITG (FT9) indicates that the most intense language-specific processing was either not occurring optimally or was less effectively distributed.

The distribution of the γ2 hubs in the control group (BC) differed significantly from that in children with DD (*χ*^2^ = 12.652; *p* = 0.0004; Table 4; Figure 2b). Control group hubs were highly centralized along the midline and included the left temporal region, indicating an efficient communication flow that routed through hubs for rapid integration of auditory information. The PreCG (C1, C2) and PstCG (Cz) acted as primary hubs for information flow between executive regions and posterior sensory areas, providing rapid motor-cognitive integration. The left aITG (FT9) served as an essential communication hub for routing phonological and auditory-linguistic information to the central processing stream. The occipital midline (Oz) mediated sensory and attentional integration, efficiently connecting the posterior attentional system to central and frontal regions.

The dyslexic group’s γ2 hubs, with higher BC, revealed an expanded, left fronto-central focus, along with the inclusion of right-sided parietal hubs. This indicated a less focused and more effortful compensatory communication strategy. High BC in the left IFG (FC5), PreCG (C2), and PstCG (Cz, C3) indicated heavy routing of communication through the left motor and articulatory systems. The left aITG (FT9) and MTG (T7) were necessary hubs for auditory input. However, their mediation role was distributed among several left-sided motor hubs, which indicated a constriction or heavy reliance on those specific hubs. Additionally, the right SPL (CP2) and IPL (P4) suggested that information flow was being routed through spatial and non-linguistic attention systems to manage the high cognitive load, a common compensatory finding in dyslexia. The γ2 network (BC) for children with DD displayed less efficient, expanded hub distribution that heavily relied on the left articulatory system (FC5, C2, Cz, C3) and right parietal compensation (CP2, P4). The information flow was less centrally focused (only Cz and C2 overlapped with controls) and instead restricted through effortful motor pathways and atypical spatial attention pathways.

### 3.6. Local Measures of the Frequency Functional EEG Networks During Auditory Discrimination of Pseudowords

#### 3.6.1. Gamma1-Frequency Network During Auditory Pseudoword Discrimination

**Figure 3:**
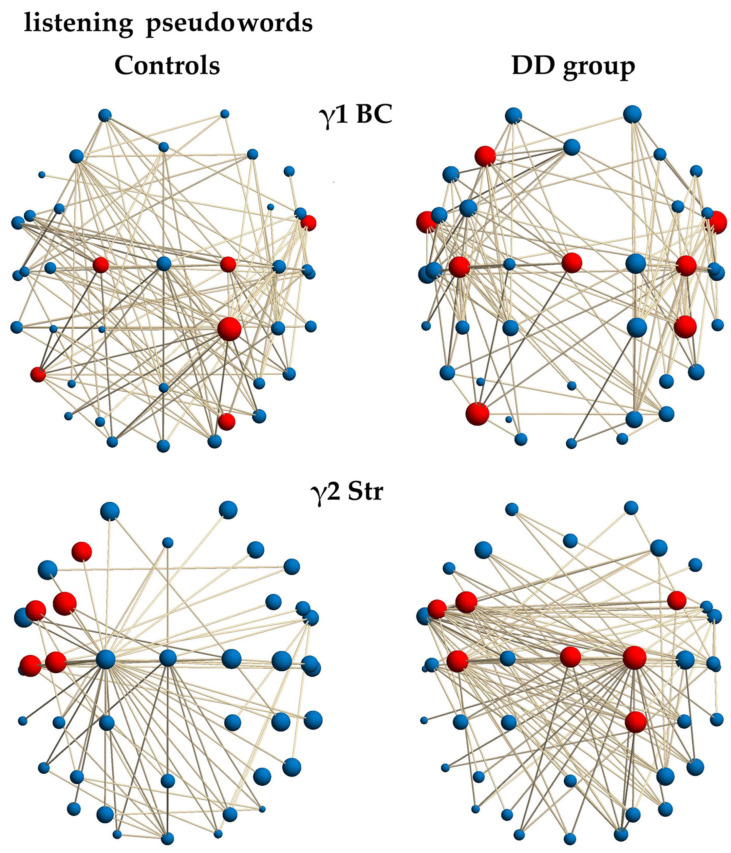
Frequency networks (main hubs in red, strong connections in black) in controls and children with DD for listening of monosyllabic pseudowords: (1) BC of hubs in γ1-network: Controls: FT10, C1–C2, P7, CP2, P04. Children with DD: F3, FT9–10, Cz, C3–4, CP4, P07. (2) Str of hubs in γ2-network: Controls: F3, FC3, FC5, C3, C5. Children with DD: FC3–4, FC5, C2, Cz, C3, CP2.

The BC hub of the γ1 network showed key differences in critical information hubs during auditory discrimination of pseudowords (BC, *χ*^2^ = 13.211; *p* = 0.0002; Table 5; Figure 3). In the γ1 network, control children relied on a balanced, sensory-integrative communication network. In contrast, children with DD used a constrained, executive-motor information flow system. The control group’s γ1 hubs were diffuse and bilateral, spanning central and posterior sensory areas. PreCG (C1–C2) connected sensory input with frontal control. Right aITG (FT10) was a right auditory bridge for non-linguistic/prosodic acoustic features. The posterior SPL (CP2), MTG (P7), and SOG (PO4) were bilateral sensory gateways, routing spatial and perceptual features crucial for binding acoustic elements.

In contrast, the hubs in the γ1 network (BC) of the DD group were more numerous, predominantly left-lateralized, and clustered in frontal, central, and temporal regions. The left MFG (F3) acted as a central executive hub, coordinating working memory and control signals, which reflects a greater reliance on conscious, effortful processing. The bilateral aITG (FT9–10) served as the main auditory bridge for phonological input, with their bilateral presence suggesting these nodes are essential and potentially overburdened. The network in children with DD was more constrained and primarily motor-dominated, with communication channeled through the articulatory loop, including the MFG (F3) and PstCG (Cz, C3–C4). Posterior hubs, such as the right IPL (CP4) and left MOG (PO7), pointed to more localized routing of spatial and attentional information, rather than global sensory integration. This pattern indicates a compensatory shift from sensory analysis to reliance on motor rehearsal.

#### 3.6.2. Gamma2-Frequency Network During Auditory Pseudoword Discrimination

For the auditory discrimination of pseudowords, the strength hubs in the γ2 frequency network indicated the brain areas most intensely and directly connected for processing the complex phonetic information. The between-groups comparison highlighted a difference in the γ2 frequency network (Str; *χ*^2^ = 8.6855; *p* = 0.0032; Table 5; Figure 3). The controls focused their maximal effort on a constrained set of highly integrated left-hemisphere frontal and central hubs. Left MFG (FC3, F3), IFG (FC5) reflected the maximal, direct resource allocation to working memory and phonological encoding (articulatory planning) necessary for rapid pseudoword analysis. Left PstCG (C3, C5) showed intense connectivity within the left motor system, supporting the precise and rapid use of inner speech for maintaining and verifying the complex phonetic sequence.

In contrast, the dyslexic group’s maximal effort in the γ2 network (Str) was distributed bilaterally and included midline and right-sided regions, reflecting a less efficient and more dispersed approach that recruited a broader range of neural resources to handle the cognitive load. Bilateral MFG (FC3–4) and left IFG (FC5) indicated executive and motor involvement across both hemispheres. Robust activation of the PreCG (C2) and PstCG (Cz, C3) highlighted reliance on a generalized, centralized effort system, which may compensate for reduced task-specific efficiency elsewhere. Engagement of the right SPL (CP2) suggested unique right posterior participation, and the involvement of a right parietal hub indicated that meeting the highest cognitive demands required tapping into spatial or non-linguistic attentional mechanisms—hallmarks of compensatory processing.

## 4. Discussion

The study found significant differences in the global metrics of the brain network of children with DD than in controls across multiple frequency bands (δ-, θ, β, and γ1), suggesting an overall less effective and less optimal network organization for cognitive processing for auditory discrimination of monosyllabic words. Significant differences in global brain network metrics emerged between children with DD and controls across delta, theta, beta, and gamma1 bands, suggesting a fundamentally less optimal network organization for the auditory discrimination of monosyllabic words. These results highlight a complex, frequency-dependent relationship between SWP and behavioral efficiency, revealing distinct “neural economy” profiles.

Typical readers exhibited an SWP, reflecting an optimal balance of integration and segregation, which facilitates efficiency up to the β1 range, where this frequency-dependent relationship was inverted at higher frequencies. This suggested that the advantages of a small-world topology were not absolute but were constrained by the computational costs of high-frequency synchronization during lexical retrieval. In contrast, the DD group exhibited an “efficiency reversal” within the δ-frequency band, and the high SWP was strongly associated with reduced processing efficiency when listening to both words and pseudowords. This persistent δ-frequency band inefficiency served as a neurobiological marker for disruptions in low-frequency networks vital for the temporal parsing of speech rhythm [77]. Such deficits in the brain with DD may reflect a “noisy” network state or an over-reliance on compensatory mechanisms that hinder rapid phonological processing, aligning with rhythmic EEG abnormalities [78,79]. While children with DD mirrored the high-frequency (β2) SWP inefficiency during word listening, the relationship reversed for pseudowords, where the higher β2 SWP suddenly predicted “enhanced efficiency”. This indicated that for children with DD, the utility of a small-world organization was highly contingent on the nature of the linguistic input. Although beta oscillations are traditionally linked to the preservation of a current cognitive state [80], they also facilitate communication through coherence during high-demand tasks [81]. Here, the shift toward beta reliance suggested that children with DD can recruit high-frequency networks to facilitate the effortful process of phonological decoding when automated lexical access is unavailable. Their brain appears to compensate for impaired “bottom-up” sensory processing, driven by dysfunctional low-frequency oscillations, by shifting toward an effortful, “top-down” reliance on high-frequency regulatory mechanisms to maintain behavioral performance.

### 4.1. Network Differences in Listening of Words

Building upon this hierarchical reorganization, the topological findings provide a structural explanation for the “efficiency reversals” observed in the behavioral data. The artificially high Small-World Propensity (ϕ) in the delta and gamma1 bands for children with DD does not represent superior processing, but rather a failure of functional specialization. Because this high ϕ is driven by a significant reduction in local clustering (ΔC), the network lacks the dense, modular processing required for fine-grained auditory discrimination. In the delta band specifically, this lack of segregation mirrors the impaired “bottom-up” sensory sampling previously discussed; without robust local hubs to process syllabic and prosodic information, the network remains in a state of high entropy or “neural noise” [78].

The paradoxical state of the θ and β networks, characterized by both reduced specialization (lower ΔC) and poor global efficiency (higher ΔL), further explains why these children struggle with rapid, coordinated communication. While typical readers utilize centralized and globally integrated hubs to mediate information flow, the DD network appears constrained. The higher ΔL suggests that despite attempts at neural integration, the increased characteristic path length between functional nodes creates a “communication lag” that impairs the speed of lexical access. This topological layout forces a reliance on a motor-compensatory network, particularly involving core language and motor areas, as a means of bypassing the inefficient auditory-to-phonological route [35,36].

These network deficits directly correspond to the literature on impaired δ-entrainment. The inability of the DD brain to flexibly entrain at the syllabic rate—manifesting here as poor network flexibility and low clustering—creates a non-optimal organization for temporal sampling [35,37]. During pseudoword discrimination, where the demand for phonological decoding is highest, the reduction in ΔC within the δ/θ bands likely represents a failure to create the necessary “auditory templates” for non-lexical stimuli. Consequently, the systemic shift toward beta-band reliance noted earlier is not just a preference, but a topological necessity; the brain must bypass the fragmented, poorly integrated low-frequency networks in favor of high-frequency mechanisms to achieve any degree of behavioral success.

This topological fragmentation directly underscores the behavioral inefficiencies observed, as the network itself impedes the rapid coordination required for fluent auditory processing. In children with DD, the recurrent failure to establish specialized local modules, quantified by a lower ΔC in the θ, β bands, provides the graph-theoretical foundation for observed deficits in phonological short-term memory and sensory-motor integration [5,31,32,44].

While typical readers utilize midline hubs of the dorsal stream (MFG, PstCG, precuneus) in α and β1 networks to integrate executive control and sensorimotor functions [68], children with DD exhibit a less globally coordinated system where the role of these midline hubs are significantly diminished [44]. The loss of these central integrating structures appears to force a “focal point of congestion” regarding executive control, sometimes centered singularly on MFC (β2, BC). While controls benefit from an optimal distribution of resources across bilateral frontal and central regions, the DD network becomes heavily left-lateralized and constrained to a tight IFG-aITG-PstCG-MTG circuit [2,47,49]. This shift represents a structural constraint and a subsequent functional overload of core language and articulatory circuits. Specifically, the dominant and isolated sensorimotor hubs in the DD group (high strength/BC in β1 and γ) indicate a compensatory over-reliance on motor planning and articulatory rehearsal, an “inner speech” strategy, to offset failing phonological processing [5,19,20,49]. These atypical hub patterns also provide a structural basis for the speech-brain entrainment deficits previously noted [35,36,38]. In the α network, which governs attention and timing, the DD group lacks the centralized integration (MFG, PstCG, precuneus) seen in controls, instead showing atypical a aITG strength and an increased sensorimotor focus [38,49]. This suggests that the attention-gating mechanisms necessary for language are fundamentally redirected. Furthermore, the loss of midline MFG and SPL hubs in the β1 network, critical for maintaining a heard word in working memory, leaves children with DD unable to utilize efficient frontal control [15,19,20]. Consequently, the dyslexic brain must rely on a constrained IFG/PstCG loop to manually “rehearse” phonetic structures, substituting effortful, motor-driven maintenance for the automated, globally integrated processing of the dorsal stream seen in typical development.

In the β2 network (Strength), children with DD shift their central hubs toward a heavily left-lateralized circuit involving the left aITG, MTG, IFG, and PstCG [2,47,49]. While typical readers maintain efficient, distributed cognitive control via bilateral MFG hubs, the DD network exhibits a singular, precarious reliance on a solitary MFG hub. This high load on the left aITG/MTG and auditory cortex reflects an overburdened, local language-motor loop that lacks the global coordination necessary for rapid word processing [49].

The γ1 frequency network, associated with high-frequency analysis necessary for tasks like phonetic feature binding and sharp acoustic encoding, showed the most critical deficits in the DD network: a breakdown of automatic feature binding. The γ1 network in DD exhibited a complete absence of posterior sensory hub, and it was strongly routed through the left articulatory/core language areas (BC: IFG, PstCG, MTG), as well as left frontal/motor dominated (Str: IFG, MFG, PstCG). The absence of posterior hubs suggested a breakdown in automatic feature binding [35,38], forcing the binding process into the effortful motor rehearsal loop [19,20,49]. This shift into a compensatory motor pathway is directly reflected in the network’s functional efficiency. The dyslexic brain relies on increased small-world organization in this high-frequency band to manage the high cognitive load of word listening, where the motor-heavy architecture is a necessary compensation for the sensory deficit. The left-dominant 30 Hz-entrainment deficit in the left auditory cortex (AC) in children with DD and the pronounced 40 Hz-entrainment in the phonological type of DD (hearing “reassembly” [35,36,38]) corresponded to the absence of sensory hubs that show the failure of the automatic phonetic feature binding mechanism [35,38]. The left IFG/PstCG dominance showed the brain compensating by intensely using the motor system to manually bind sound features, aligning with the γ1-entrainment deficit [38]. The control group’s γ1 network (feature binding) showed robust bilateral posterior sensory hubs (e.g., left IPL, right SOG, OL), reflecting an automatic, sensory-driven grouping of acoustic features [24,35]. This architecture facilitates an automatic, sensory-driven grouping of acoustic features, providing the precision necessary to capture the sharp, distinct high-frequency cues required for consonant discrimination. Unlike the compensatory strategies observed in dyslexia, the control group’s gamma network achieves high performance through these specialized sensory pathways without requiring an extensive, motor-dependent small-world organization, and maintains a specialized, streamlined sensory route that bypasses the need for effortful motor-system integration.

The γ2 network is associated with peak cognitive effort and conscious perception. The γ2 network (Str/BC) in children with DD showed an atypical right-hemisphere and posterior focus, with strength (Str) hubs located in the MFG, PstCG, SPL, and IPL [49]. BC hubs in this network were notably motor-heavy (right IFG, PreCG, and left PstCG) and utilized right parietal compensation (Str/BC: SPL, IPL) [49]. Under high cognitive demand, the brain hubs of children with DD redirected maximal effort toward these atypical right-sided spatial/attentional areas (MFG, IPL) to manage word listening [18,49]. This reflects an inefficient, unspecialized strategy that compensates for a lack of typical left-hemisphere specialization [47,49]. This atypical right parietal compensation is expanded through motor mediation. Consequently, intense cognitive loads are managed by these right-sided resources and a highly localized, inefficient articulatory system [19,20,49]. A shift towards higher-frequency γ2-entrainment in the AC has been observed in children with visual dyslexia [18,38]. In the γ2 network of the controls, intense left language load (IFG, aITG) [27,31,32] was supported by bilateral sensorimotor strength. The γ2 network (BC) was focused on the efficient midline/left temporal route [30,49].

These functional networks suggested that the word listening is significantly more effortful and inefficient for children with DD, requiring them to route crucial information through alternative, often motor-based pathways [19,20,49].

The persistent overreliance on the motor/articulatory system (left PstCG, IFG) in the word listening task is a topological manifestation of the phonological deficit [15,19,20,82]. This aligns perfectly with the phonological deficit hypothesis, which postulates that the core problem in dyslexia is a difficulty in representing and manipulating the sounds of language [3,15].

The evidence for network organization in DD demonstrates a failure of the automatic phonetic feature binding mechanism [35,38]. This explains why high-frequency components, which are crucial for distinguishing Bulgarian consonants, were not efficiently captured [51]. Bulgarian consonants require the auditory system to be highly precise in capturing distinct high-frequency cues. The DD brain’s failure in γ-band feature binding means it cannot achieve this required sharpness of encoding [35,38]. The acoustic cues for “front-vowel” vs. “back-vowel” consonants are likely merged or processed too slowly, leading to phonological confusion [4]. The network demonstrated that the failure to process sharp, high-frequency acoustic details (γ band) cascades down to compromise the function of the low-frequency bands (δ/θ), creating a unified, systemic deficit in temporal speech processing [35,36,49].

### 4.2. Network Differences in Listening to Pseudowords

The behavioral findings showed that the consistent inefficiency observed in the δ band across both conditions suggested a core deficit in low-frequency networks, which were critical for speech rhythm parsing. However, the ‘efficiency crossover’ observed in the β band during pseudoword processing provided evidence of a compensatory mechanism. It appears that while the brains of children with DD struggle with automated lexical access, they can recruit high-frequency networks to improve efficiency during effortful phonological decoding. This indicated that DD is characterized by a shift from inefficient, low-frequency ‘bottom-up’ processing to a more demanding, high-frequency ‘top-down’ reliance.

When processing pseudowords, the DD network consistently showed reduced functional segregation (lower ΔC) across multiple bands (θ, β). This suggested a core deficit in establishing and utilizing specialized, highly clustered local brain modules. The networks of controls, however, demonstrated significantly higher functional segregation (ΔC), reflecting a more mature system capable of effective specialized processing. The networks of children with DD, however, showed network compromises, either by shaving poorly specialized local modules (low ΔC) or, in the γ2 band, by employing a shorter, less complex routing strategy (lower ΔL). The functional topology in dyslexia was less optimally balanced for specialized local processing and efficient long-range communication, a requirement for successful reading and language decoding [3,14].

Auditory processing monosyllabic pseudowords (novel, non-lexical stimuli) required greater reliance on phonological decoding [4,11] and efficient module specialization. The findings revealed a recurrent deficit in local efficiency for the DD group. In θ- and β2-networks, both groups had a similar path length (ΔL, or global efficiency), but the controls had a significantly higher ΔC (clustering) than the children with dyslexia. The controls possessed a more efficiently segregated network (higher ΔC), meaning their local brain regions were more specialized and tightly clustered, which is a hallmark of healthy, efficient brain function. The DD group, conversely, showed a less specialized local network (lower ΔC, local segregation deficit) [44], suggesting impaired local efficiency or processing within functional brain modules, despite having comparable global efficiency (similar ΔL) to the controls.

In the β1-network, similar to θ- and β2, the primary difference was a reduction in functional segregation (lower ΔC) in the DD group, with preserved global measures (ΔL and ϕ were similar between groups) [43,44,67]. This confirmed a failure in establishing specialized, efficient local modules crucial for specific cognitive tasks, particularly in the frequency band relevant for sensory-motor integration [31,32,49]. In the γ2-network, both groups had similar ΔC (local processing preserved). The DD group showed a lower ΔL (more global efficiency) than the control group. Therefore, the network of controls utilized a more complex, longer-path routing for information processing in this high-frequency band, reflecting more sophisticated cognitive integration [44,49]. The lower ΔL in the DD network may reflect a shorter, less complex, or less mature routing strategy that artificially appears more efficient in the graph metric but signifies a less sophisticated functional organization [44,49].

The network analysis across the α, β, and γ frequency bands revealed that children with DD process novel auditory-linguistic input (pseudowords) using a less efficient, motor-compensatory, and constrained functional network organization compared to control children [49].

The evidence in the β1, γ1, γ2 networks for the strategy substituting efficient auditory decoding with motor simulation was the involvement of left IFG/PstCG cortices [49], which serve as dominant strength hubs (maximal effort) and BC hubs (information bottleneck) to rehearse and bind the complex, novel phonetic features of the pseudoword [19,20,22,49]. The γ1 network in DD failed to utilize the efficient, distributed sensory pathways for integrating acoustic information [35,38]. In the γ1 network (Str/BC), the controls relied on posterior sensory hubs (right SOG, central OL) for fast, sensory-driven binding [35]. These hubs were absent in the γ1 network of the children with DD [38,49]. The γ1 network in DD failed to utilize the efficient, distributed sensory pathways for integrating acoustic information [35,38]. In the α network (BC), the controls used a central, integrated dorsal stream (central PstCG, precuneus) for global mediation, While the α network for children with DD used a singular, constrained mediation [49], routed through the left IFG/PstCG loop [19,20,49], significantly increasing the temporal and cognitive cost of discrimination of pseudowords.

The DD network’s response to high cognitive load (β2 and γ2) revealed an inefficient expansion of resources [49]. The control γ2 network (Str) was efficient and specialized, focusing on the left linguistic loop (left MFG, PstCG) [31,32] to maximize resource concentration in the specialized system. The γ2 (Str) of the dyslexics had a compensatory and dispersed role, characterized by expanded bilateral recruitment (right MFG, SPL) [49]. This shift distributed peak effort to right-hemisphere executive/spatial areas, a common sign of compensatory reliance on non-linguistic resources [18,49]. The β2 network (BC) for the controls was integrated (central MFG and bilateral posteriorly), indicating that the top-down control was globally routed. The group with DD was constrained with the central MFG hub serving as a singular mediator [31,32,49]. Control and routing functions were concentrated and potentially bottlenecked.

The recurrent failure in δ, β networks to establish specialized local modules (low ΔC) provided the graph measure foundation for the specific regional/frequency deficits [38,49]. The reduced ability to cluster (segregate) locally was the structural compromise underlying deficient phonological working memory [15,19,20] and sensory-motor integration (relevant to β) [31,32,49]. The finding that the phonological type of dyslexia (β) showed insufficient low-frequency β changes over the IFG and IPL, correlating with worse phonological short-term memory [15,19,20,38].

In the γ2-frequency network of children with DD, a lower ΔL (shorter path length) supports the hypothesis that the DD network utilizes a less sophisticated functional organization. Furthermore, the high γ-entrainment in the non-phonological type of DD [38] suggested a compensatory or disorganized auditory timing mechanism (e.g., too fast phonemic sampling) [35], which can lead to subsequent verbal memory deficits [38]. Therefore, a deficit in the left planum temporale (PT) disproportionately affected the repetition of pseudowords compared to familiar words [6], further supporting the PT’s role in rapid decoding. Since pseudowords must be processed solely based on their constituent speech sounds (phonemes) rather than accessing existing lexical-semantic knowledge [10,11], the left PT has a crucial intermediary role in converting raw acoustic data into meaningful linguistic units [6,21,27].

## 5. Limitations

While the present study focused exclusively on task-evoked functional connectivity to isolate the mechanisms of speech discrimination, future research incorporating resting-state EEG could offer a valuable baseline comparison. Analyzing intrinsic connectivity at rest would help determine whether the observed network differences in children with DD are unique to active language processing or reflect a more fundamental divergence in baseline neural architecture. The current findings provide insights into the neural efficiency of dyslexia within a transparent orthography. While the low-frequency δ sampling deficits and reduced global integration (∆L) appear to be universal neurobiological markers of DD, the specific recruitment of the IFG/PstCG ‘articulatory loop’ may reflect a compensatory strategy optimized for the sub-lexical decoding requirements of shallow orthographies. Future cross-linguistic studies should examine whether the ‘efficiency reversal’ observed in the β2 band remains a stable predictor of performance in deep orthographies, where decoding accuracy, rather than just speed, remains a primary challenge.

## 6. Conclusions

The dyslexic network exhibited a pervasive pattern of inefficiency and high cognitive cost during the auditory discrimination of words and pseudowords. A high SWP (ϕ) does not inherently equate to functional optimality. In the DD group, the observed configuration, characterized by short path lengths but reduced local specialization (∆C), suggests a ‘shortcut’ strategy. The network could not establish optimal communication pathways (BC), resulting in constrained information flow and potential bottlenecks. The topology bypassed specialized linguistic modules in favor of atypical motor-heavy pathways, a configuration that the regression models confirmed was behaviorally inefficient and cognitively taxing [75]. A critical question remains whether the observed network alterations represent primary neurobiological markers of dyslexia or secondary adaptations to reduced reading experience. Our data suggest a hybrid model. The persistent low-frequency (δ) network ‘noise’ serves as a primary deficit in temporal sampling, which remains stable regardless of task condition. Conversely, the recruitment of the β2 motor-linguistic loop (IFG/PstCG) during pseudoword processing appears to be a secondary compensatory adaptation. This adaptation allows the dyslexic brain to achieve phonetic decoding through an ‘articulatory-rehearsal’ strategy, effectively bypassing the primary phonological bottleneck, albeit at a higher topological and cognitive cost. The system compensated by over-engaging the articulatory loop (high strength in left IFG/PstCG) and recruiting atypical right-sided resources under peak load (γ). This specific functional signature, characterized by poor global routing and motor-heavy compensation, underpinned the difficulty DD children had in rapid and accurate novel word decoding, which is fundamental to reading acquisition.

## Figures and Tables

**Table 1:** Three frequency group ranges based on a Bulgarian Frequency Dictionary.

Frequency Group	Rank Range	Frequency Range (Numerator/Denominator)
High	46 to 110.5	387/5 to 138/5
Average	150 to 273	88/5 to 40/5
Low	368.5 to 624.5	13/3 to 25/5

**Table 2:** Global measures (P: ϕ, ΔC, ΔL) of the frequency networks (δ, θ, α, β1, β2, γ1, γ2) are presented. Statistically significant differences between groups for the corresponding parameters are marked (significance threshold *p* < 0.017; φ, ∆C, ∆L) in the auditory discrimination.

f	P	Controls	Children with DD	Controls/	Children DD
		Mean ± s.e.	Mean ± s.e.	*p*	*χ* ^2^
δ	ϕ	0.5609 ± 0.0137	0.6128 ± 0.0128	**0.0112**	6.4295
	ΔC	0.5081 ± 0.0229	0.4289 ± 0.0199	**0.0103**	6.5818
	ΔL	0.2701 ± 0.0207	0.2673 ± 0.0168	0.7575	0.0953
θ	ϕ	0.5021 ± 0.0104	0.5687 ± 0.0108	**1.1 × 10^−5^**	19.289
	ΔC	0.6875 ± 0.0157	0.5625 ± 0.0180	**1.3 × 10^−6^**	23.449
	ΔL	0.1176 ± 0.0065	0.1667 ± 0.0092	**1.7 × 10^−5^**	18.505
α	ϕ	0.5268 ± 0.0099	0.5425 ± 0.0105	0.406	0.6901
	ΔC	0.6198 ± 0.0187	0.6131 ± 0.0175	0.7709	0.0847
	ΔL	0.1658 ± 0.0101	0.1358 ± 0.0074	0.0171	5.6783
β1	ϕ	0.4567 ± 0.0087	0.5032 ± 0.0081	**9.1 × 10^−5^**	15.314
	ΔC	0.7621 ± 0.0129	0.6834 ± 0.0135	**5.3 × 10^−5^**	16.345
	ΔL	0.0689 ± 0.0042	0.0996 ± 0.0064	**0.0011**	10.573
β2	ϕ	0.4589 ± 0.0093	0.5046 ± 0.0089	**0.0001**	14.028
	ΔC	0.7558 ± 0.0145	0.6817 ± 0.0144	**9.3 × 10^−5^**	15.272
	ΔL	0.0742 ± 0.0051	0.1006 ± 0.0063	**0.0005**	11.931
γ1	ϕ	0.4853 ± 0.0089	0.5258 ± 0.0095	**0.0036**	8.4308
	ΔC	0.6966 ± 0.0161	0.6291 ± 0.0169	**0.0043**	8.1416
	ΔL	0.1279 ± 0.0095	0.1433 ± 0.0091	0.2019	1.6279
γ2	ϕ	0.4551 ± 0.0113	0.4807 ± 0.0113	0.1714	1.8699
	ΔC	0.5821 ± 0.0243	0.5144 ± 0.0236	0.0908	2.8596
	ΔL	0.3431 ± 0.0225	0.3440 ± 0.0213	0.7133	0.1349

**Table 3:** Global measures (P: ϕ, ΔC, ΔL) of the frequency networks (f: δ, θ, α, β1, β2, γ1, γ2) are presented. Statistically significant differences between groups for the corresponding parameters are noted (significance threshold *p* < 0.017) in auditory discrimination of pseudowords.

f	P	Control	Children with DD	Controls/	Children DD
		Mean ± s.e.	Mean ± s.e.	*p*	*χ* ^2^
δ	ϕ	0.6056 ± 0.0177	0.6300 ± 0.0143	0.1741	1.8465
	ΔC	0.4462 ± 0.0280	0.3961 ± 0.0228	0.1222	2.3887
	ΔL	0.2576 ± 0.0214	0.2644 ± 0.0169	0.7063	0.1419
θ	ϕ	0.5233 ± 0.0117	0.5672 ± 0.0112	**0.0162**	5.7753
	ΔC	0.6369 ± 0.0215	0.5685 ± 0.0187	**0.0111**	6.4371
	ΔL	0.1413 ± 0.0094	0.1582 ± 0.0082	0.0861	2.9449
α	ϕ	0.5502 ± 0.0113	0.5299 ± 0.0100	0.1425	2.1498
	ΔC	0.5893 ± 0.0198	0.6404 ± 0.0158	0.0434	4.0785
	ΔL	0.16362 ± 0.011	0.1253 ± 0.0062	**0.0069**	7.2961
β1	ϕ	0.4609 ± 0.0096	0.4878 ± 0.0085	0.0222	5.2285
	ΔC	0.7537 ± 0.0149	0.7078 ± 0.0136	**0.0131**	6.1522
	ΔL	0.0733 ± 0.0049	0.0897 ± 0.0062	0.0764	3.139
β2	ϕ	0.4626 ± 0.0103	0.5091 ± 0.0087	**0.0005**	12.051
	ΔC	0.7516 ± 0.0156	0.6823 ± 0.0133	**0.0004**	12.344
	ΔL	0.0737 ± 0.0049	0.0875 ± 0.0042	0.0292	4.751
γ1	ϕ	0.5004 ± 0.0111	0.5308 ± 0.0090	0.0424	4.1163
	ΔC	0.6760 ± 0.0183	0.6294 ± 0.0146	0.0395	4.2373
	ΔL	0.1252 ± 0.0118	0.1366 ± 0.0086	0.1445	2.1293
γ2	ϕ	0.465 ± 0.0131	0.5172 ± 0.0125	0.0183	5.566
	ΔC	0.5198 ± 0.0273	0.4762 ± 0.0233	0.3173	0.9998
	ΔL	0.3857 ± 0.0269	0.3152 ± 0.0199	**0.0142**	6.0045

**Table 4:** Local characteristics of hubs in auditory discrimination of monosyllabic words of 3–4 letters. Statistically significant differences between groups for the corresponding parameters are noted (significance threshold *p* < 0.025) in auditory discrimination of pseudowords.

f	P	Controls	Children with DD	Controls/	Children DD
		hubs	hubs	*p*	*χ* ^2^
δ	str	FC3 FC5 Cz C3 C6 CP1 P8	AF3 Fz F3 FC3 F7 FC5 C3 CP1 T7	0.5456	0.3652
	BC	F7 C3 P3 P04 P8 P08 O2	FC3 FT9–10 C3 P08 T7	0.4581	0.5505
θ	str	AF3 F3 FC3 FC5 T8	AF3 F3 FC3 F7 FC5 C3 C5	0.7104	0.1378
	BC	Fz F3 FC4 FT9 P04 P8	AF3 FT9 C3 P7 P07-P08	0.5967	0.2799
α	str	Fz Cz C3 C5 CP1 Pz P08	FT9 Fz C1 Cz C3 C5 P03–04 O2	**0.0134**	6.114
	BC	Fz FT9 CP2 P04 P08	Fz FT9 C3 P04 P08 P8 O2	0.4865	0.4842
β1	str	Fz F4 Cz C5 CP2 Oz	C3 C5 CP1–2 CP4	**4.57 × 10^−6^**	21.01
	BC	Fz FT9–10 Cz Oz O2	FC5 FT9 Cz C3 C5 T7 P07-P08 Oz	**0.0014**	10.192
β2	str	F3–4 F7 C3–4 P08 O1	Fz FC5 FT9 C2 C5 CP1 CP3 T7	**4.03 × 10^−7^**	25.678
	BC	F8 FT10 C4 P08 P8 O1	Fz FT9–10 C2 C5 T7	**0.0003**	12.994
γ1	str	F4 Cz C4 CP3 P04 Oz	FC3 FC5 C3 C5	**0.0003**	13.352
	BC	C4 P04 Oz	FC5 FT10 C3 T7	**0.0017**	9.768
γ2	str	FC3 FC5 FT9 C2 Cz C3–C4 C5	FC4 C2 Cz C4 CP2 P4	**4.3 × 10^−11^**	43.463
	BC	FT9 C1–2 Cz Oz	FC5 FT9 C2 Cz C3 CP2 P4	**0.0004**	12.652

**Table 5:** Local characteristics of hubs in auditory discrimination of monosyllabic pseudowords (of 3–4 letters). Statistically significant differences between groups for the corresponding parameters are noted (significance threshold *p* < 0.025).

F	P	Controls	Children DD	Controls/	Children DD
		hubs	hubs	*p*	*χ* ^2^
δ	str	F3–4 Fz C3 CP1 T7 O2	AF4 Fz F4 FC3 FC5 F7 C3	0.0323	4.577
	BC	FT10 P08 T7 O1–2	AF4 FC3 FC5 C6 P04 O2	0.4490	0.5729
θ	str	AF4 Fz F4 FC3 F8 C6 TP8	Fz F7 FC3 FT9 C3 C5	0.8497	0.0358
	BC	Fz F8 FT9 P04 P7 P08 TP8	Fz FT9 C3 P07–08	0.8057	0.0604
α	str	CP2 CP4 P4 P04 P8	FC3 C3 C5 CP1–2 P4 P04	0.3203	0.9875
	BC	TP8 P04 P8 P08 Oz O1–2	F7 C3 TP7 P04 P7 P08	0.319	0.9930
β1	str	F3 FC3 FC5 F7 C3 C5 CP4	FC3 FC5 FT9 C3 C5 CP1 P3	0.2248	1.4735
	BC	F7 FC3 FT9 T7	FC3 F7 FT9–10 Cz C3 T7 P04 P08 O1	0.4948	0.4659
β2	str	FC5 FT10 C3 CP2 CP4	FC3–4 C3 C5 T7	0.4691	0.5239
	BC	AF3–4 FC5 FT10 C3	C3 T7 P8	0.0439	4.0593
γ1	str	F3–4 FC6 CP2 P4 P04	AF4 C3 CP2 CP4	0.0461	3.9778
	BC	FT10 C1–C2 P7 CP2 P04	F3 FT9–10 Cz C3–4 CP4 P07	**0.0002**	13.211
γ2	str	F3 FC3 FC5 C3 C5	FC3–4 FC5 C2 Cz C3 CP2	**0.0032**	8.6855
	BC	C1 Cz	FT9 C2 Cz C3	0.1582	1.9914

## Data Availability

Access to the datasets used in this study is restricted to protect participant privacy and comply with ethical guidelines. However, researchers interested in utilizing the data may submit a reasonable request to the corresponding author.

## Supplementary Materials

The following supporting information can be downloaded at: https://www.mdpi.com/article/10.3390/neurosci7010021/s1, Figure S1: Frequency contents of words and correspondence pseudoword; Figure S2: Psychological scores of the groups (DDE-2 and Raven’s test) in standardized scores (mean ± s.d.); Figure S3: Psychological scores of the groups (Psychometric and Girolami-Boulinier tests) in standardized scores (mean ± s.d.); Figures S4–S6 and Tables S1–S4: Regression models.
