# Supplementary material for: Functional Networks in Developmental Dyslexia: Auditory Discrimination of Words and Pseudowords
Source: NeuroSci. 2026 Feb 3;7(1):21. doi: 10.3390/neurosci7010021 (PMC12921797; doi:10.3390/neurosci7010021)

Figures S1. Frequency contents of words (odd row) and under each word, correspondence pseudoword (even row).

блок (blok)

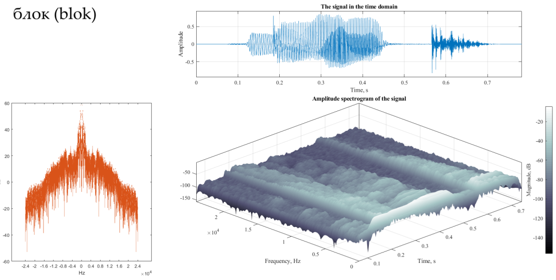

блик (blik)

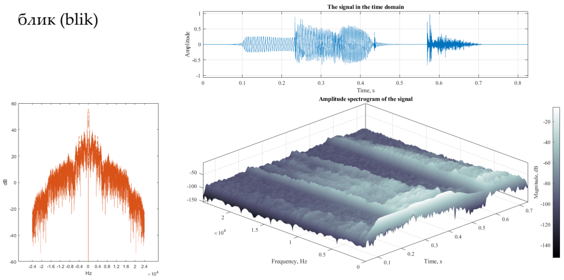

цял (tsyal)

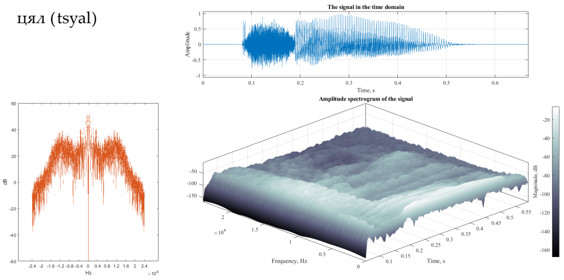

цул (tsul)

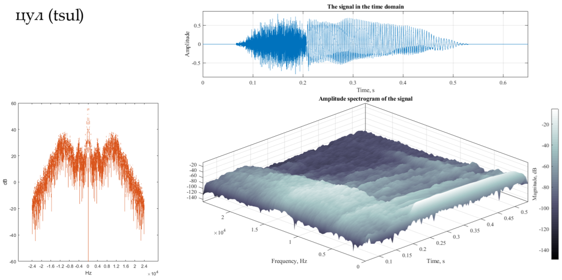

две (dve)

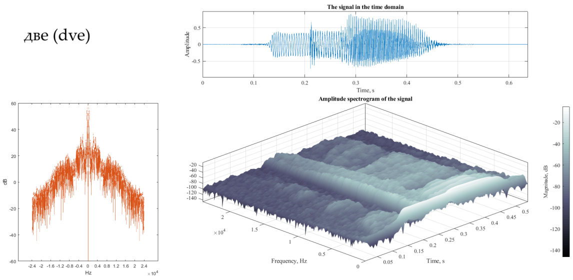

дви (dvi)

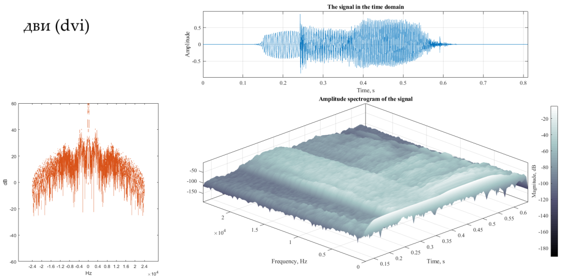

чист (chist)

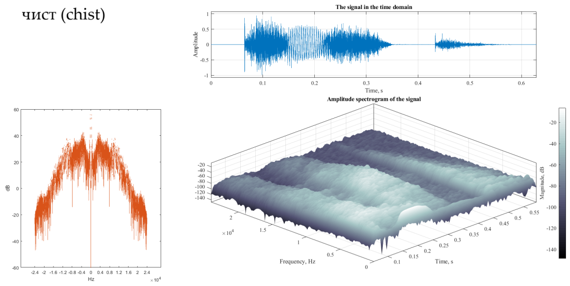

чуст (chust)

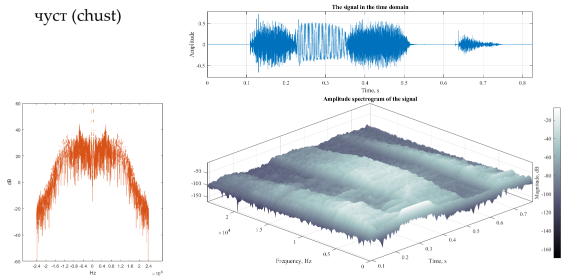

дам (dam)

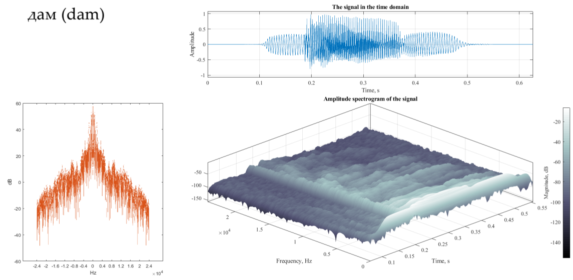

дъм (dŭm)

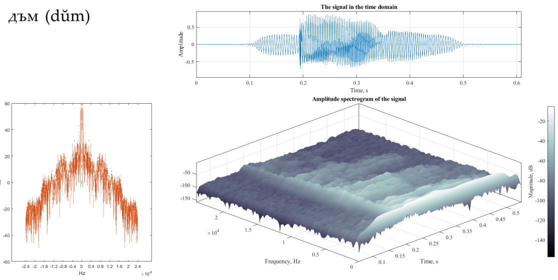

филм (film)

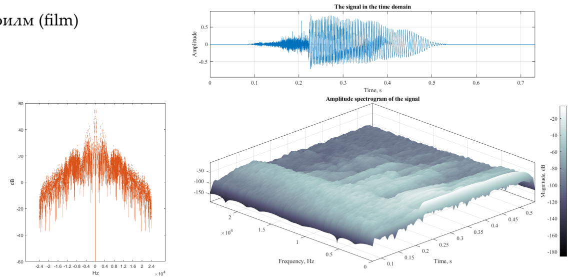

фълм (f'lm)

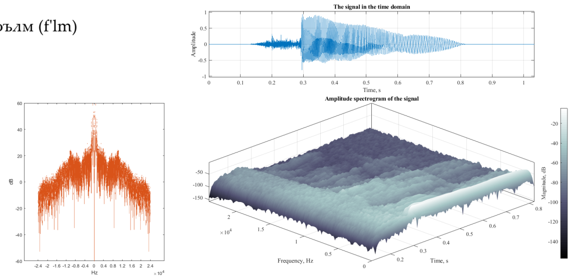

гост (gost)

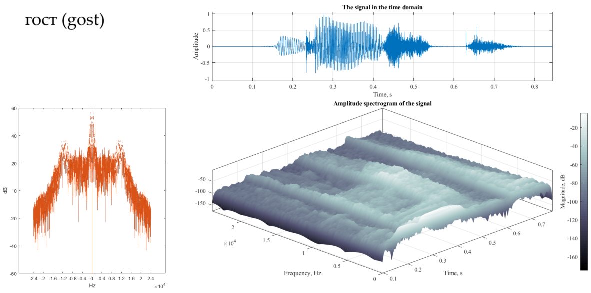

ям (yam)

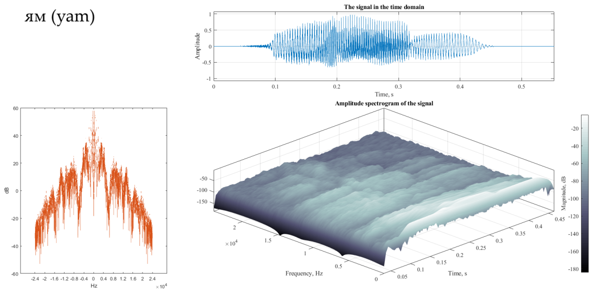

гаст (gast)

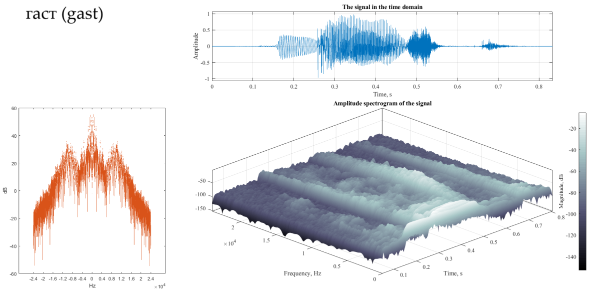

им (im)

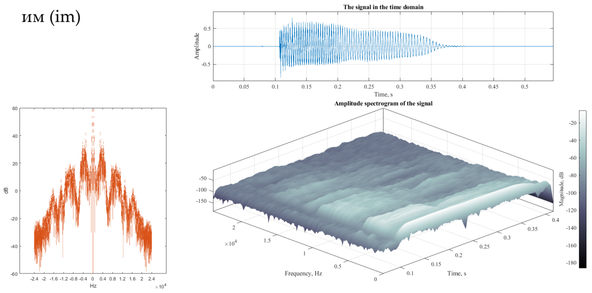

клас (klas)

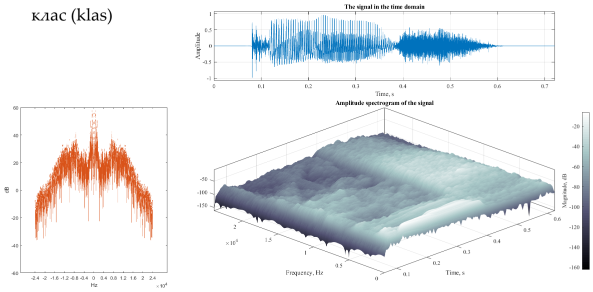

крем (krem)

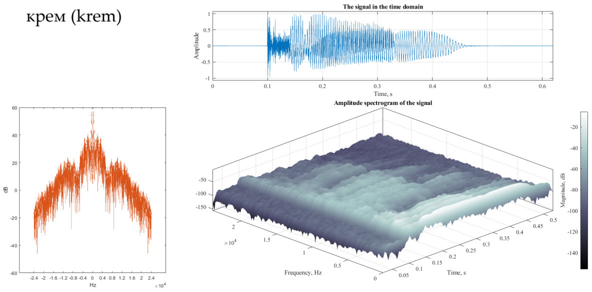

клус (klus)

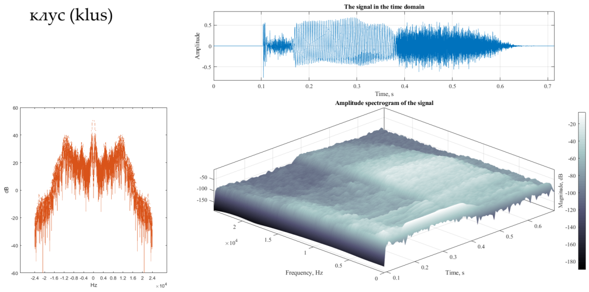

крам (kram)

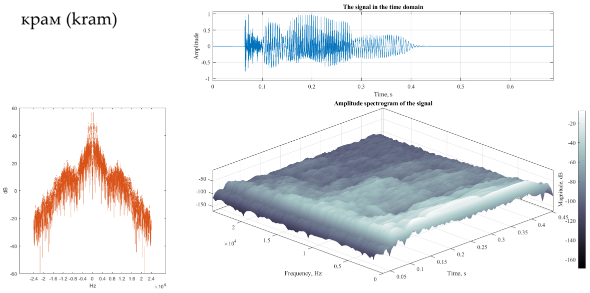

Крум (Krum)

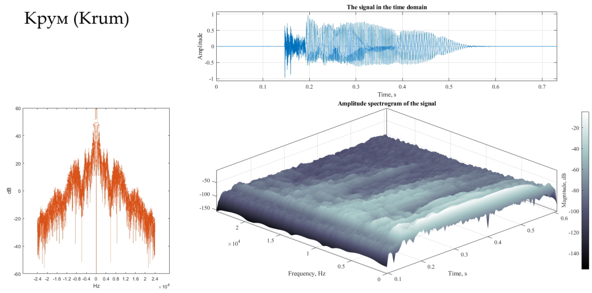

лист (list)

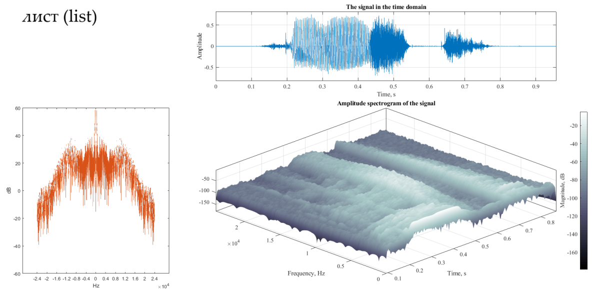

Кръм (Krŭm)

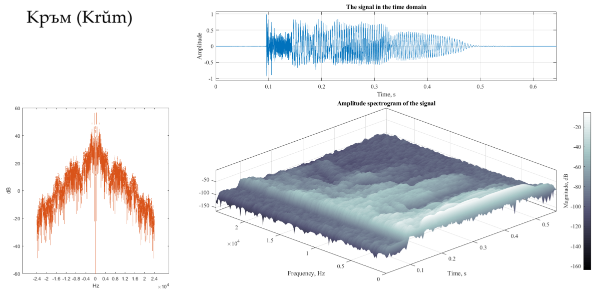

лест (lest)

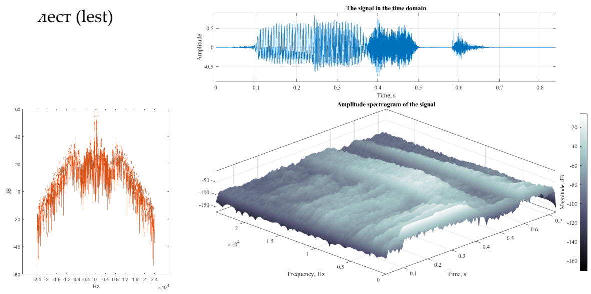

пак (pak)

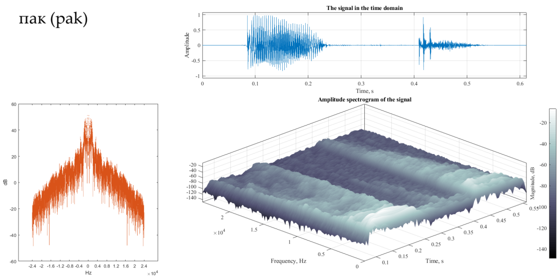

прав (prav)

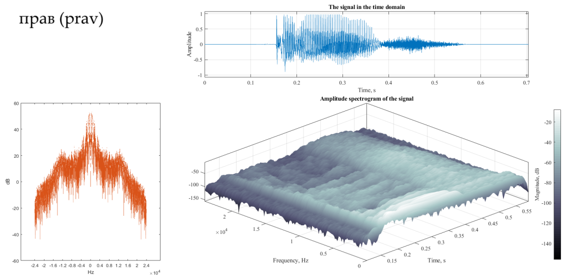

пок (pok)

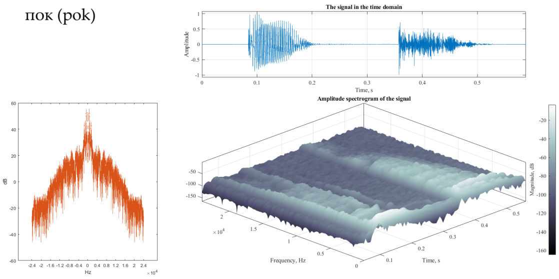

прув (pruv)

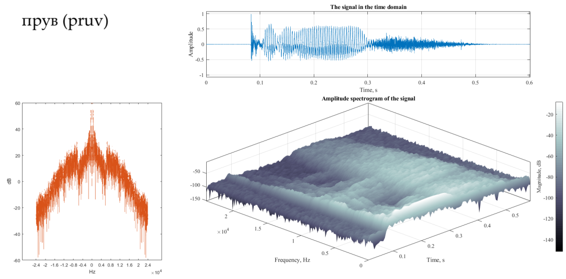

през (prez)

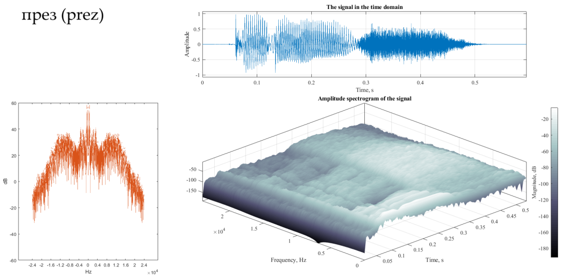

рейс (reĭs)

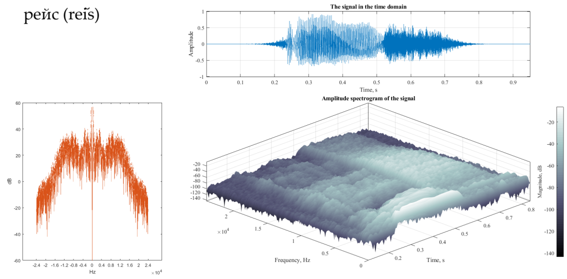

пруз (pruz)

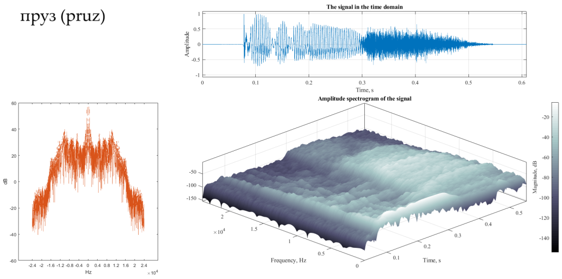

руйс (rujs)

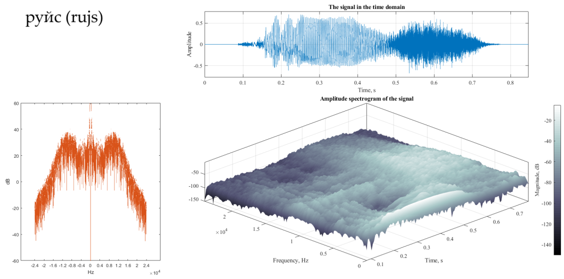

щом (shtom)

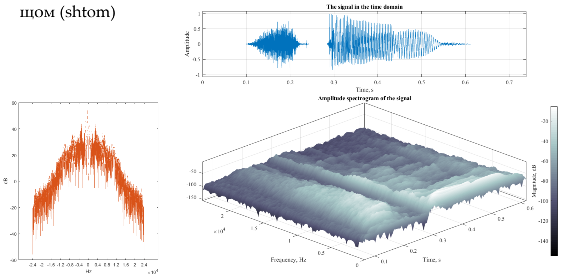

Спас (Spas)

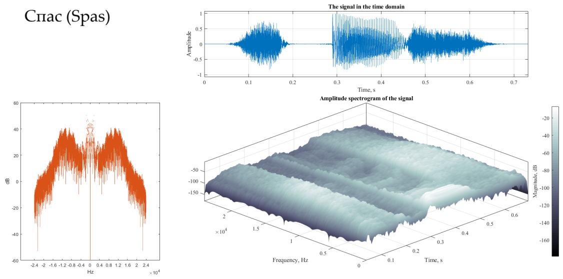

щъм (sçm)

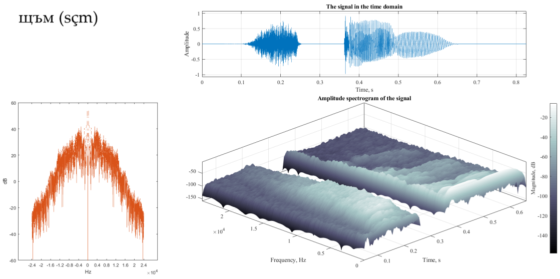

Спес (Spes)

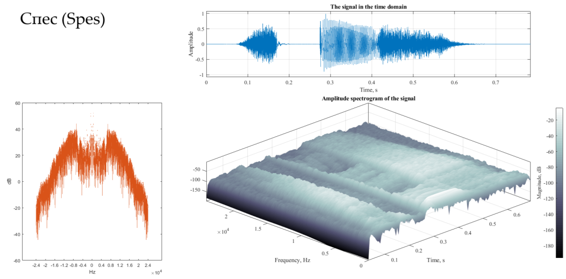

спя (spya)

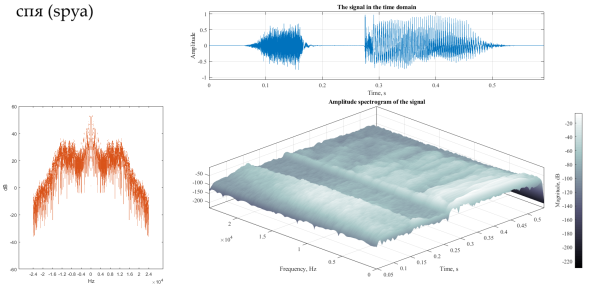

твой (tvoї)

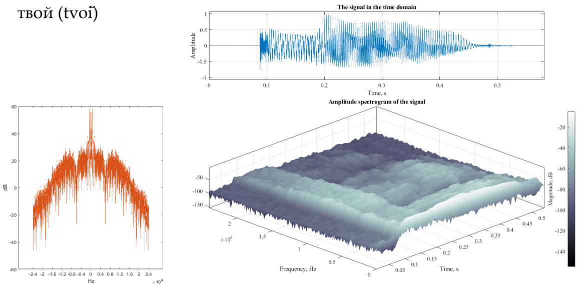

спо (spo)

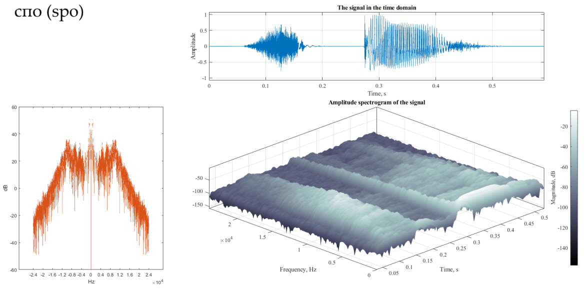

твей (tvei)

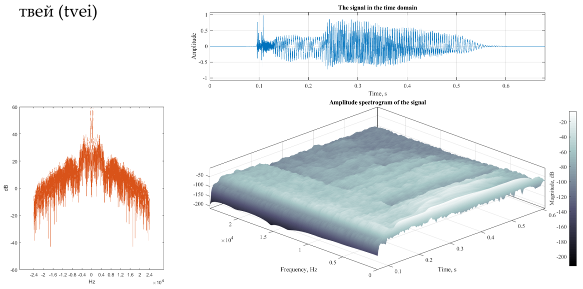

Supplement: Supplementary file 1 [file neurosci-07-00021-s001.zip › Supp_Figures_S1_words pseudowords frequency–content.pdf]
