# Supplementary material for: Functional Networks in Developmental Dyslexia: Auditory Discrimination of Words and Pseudowords
Source: NeuroSci. 2026 Feb 3;7(1):21. doi: 10.3390/neurosci7010021 (PMC12921797; doi:10.3390/neurosci7010021)
Supplement: Supplementary file 1 [file neurosci-07-00021-s001.zip › Tables_S1_S4.pdf]

Tables (S1-S4). Linear regression models (robust fit) evaluating the impact of the Inverse Efficiency Score (IES)—a behavioral metric combining speed and accuracy—and Small-World Propensity (SWP) on performance. SWP is a global measure of network topology analyzed across various spectral bands. Models are presented for Control and Dyslexia (DD) groups under Word and Pseudoword conditions.
Efficiency and Functional Network Topology for listening to words (Table S1: Controls; Table S3: DD) and pseudowords (Table S2: Controls; Table S4: DD):

Efficiency and Functional Network Topology for listening to words (Table S1 – controls, Table S3 – DD) and pseudowords (Table S2 – controls, Table S4– DD): Predictor (x1): The independent variable used in the model, including the Inverse Efficiency Score (IES) and spectral power density across specific frequency bands: Delta (δ), Theta (θ), Alpha (α), Beta (β1, β2), and Gamma (γ1, γ2); IES (Success): Inverse Efficiency Score calculated as a measure of processing speed and accuracy (RT/PC); SWP: Symbolic working memory power; Statistics: Reported values include the Intercept, Slope Estimate (regression coefficient), Standard Error (SE), t-Statistic, and p-Value; Model fit: R^2 represents the coefficient of determination, with Adjusted R^2 ($AdjR^2$) provided in parentheses.
Significance: Statistical significance is denoted as * p < 0.05, ** p < 0.01, and *** p < 0.001.

Table S1. Control group: IES vs SWP, listening to words

Predictor (x1)	Intercept	Slope (Est.)	SE (Slope)	t-Stat	p-Value	R^2 ($AdjR^2$)
IES (Success)	2557.1	-1632.6	197.70	-8.26	< 0.001***	0.688 (0.679)
SWP (δ)	1619.2	-948.57	364.08	-2.61	0.013*	0.166 (0.143)
SWP (θ)	1628.4	-1007.3	321.10	-3.14	0.003**	0.224 (0.202)
SWP (α)	1656.5	-1080.4	325.17	-3.32	0.002**	0.255 (0.235)
SWP (β1)	451.0	+1277.3	327.39	3.90	< 0.001***	0.326 (0.308)
SWP (β2)	364.38	+1533.5	337.50	4.54	< 0.001***	0.371 (0.354)
SWP (γ1)	545.55	+1031.3	313.41	3.29	0.002**	0.284 (0.264)
SWP (γ2)	428.31	+1302.6	178.10	7.31	< 0.001***	0.626 (0.616)

Table S2. Control group: IES vs SWP, listening to pseudowords

Predictor (x1)	Intercept	Slope (Est)	SE (Slope)	t-Stat	p-value	R^2 ($AdjR^2$)
IES (Success)	2980.1	-2042.5	215.6	-9.47	< 0.001***	0.708 (0.700)
SWP (δ)	1727.4	-884.76	340.7	-2.60	0.013*	0.160 (0.137)
SWP (θ)	1595.3	-931.29	452.6	-2.06	0.047*	0.172 (0.150)
SWP (α)	1451.1	-700.69	308.5	-2.27	0.029*	0.296 (0.277)
SWP (β1)	291.09	1719.6	424.2	4.05	< 0.001***	0.331 (0.313)
SWP (β2)	408.13	1366.3	332.8	4.11	< 0.001***	0.369 (0.352)
SWP (γ1)	1691.4	-1129.2	406.0	-2.78	0.008**	0.236 (0.215)
SWP (γ2)	1630.7	-1005.2	308.4	-3.26	0.002**	0.299 (0.280)

Table S3 Group with Dyslexia: IES vs SWP, listening to words

Predictor (x1)	Intercept	Slope (Est.)	SE (Slope)	t-Stat	p-Value	R^2 ($AdjR^2$)
IES (Success)	3185.6	-2452.9	274.57	-8.93	< 0.001***	0.652 (0.644)
SWP (δ)	287.27	+1772.3	546.21	3.24	0.002**	0.211 (0.193)
SWP (θ)	2305.4	-1524.8	543.34	-2.81	0.007**	0.157 (0.137)
SWP (α)	1966.0	-1021.6	472.32	-2.16	0.036*	0.104 (0.083)
SWP (β1)	2356.5	-1951.1	586.35	-3.33	0.002**	0.216 (0.197)
SWP (β2)	308.13	+2089.1	490.47	4.26	< 0.001***	0.329 (0.313)
SWP (γ1)	2256.2	-1509.5	497.88	-3.03	0.004**	0.176 (0.157)
SWP (γ2)	1998.7	-1121.9	454.55	-2.47	0.018*	0.128 (0.107)

Table S4. Group with Dyslexia: IES vs SWP, listening to pseudowords

Predictor (x1)	Intercept	Slope (Est.)	SE (Slope)	t-Stat	p-Value	R^2 ($AdjR^2$)
IES (Success)	2928.2	-2019.2	296.78	-6.80	< 0.001***	0.577 (0.564)
SWP (δ)	532.46	+1428.4	317.09	4.50	< 0.001***	0.377 (0.359)
SWP (θ)	2117.4	-1214.1	419.00	-2.90	0.007**	0.202 (0.179)
SWP (α)	1949.1	-868.63	345.60	-2.51	0.017*	0.157 (0.132)
SWP (β1)	928.02	+1018.6	475.77	2.14	0.040*	0.119 (0.093)

SWP (β2)	2400.1	-1874.2	515.11	-3.64	< 0.001***	0.281 (0.259)
SWP (γ1)	2300.1	-1562.3	341.12	-4.58	< 0.001***	0.394 (0.376)
SWP (γ2)	1872.4	-728.79	329.28	-2.21	0.034*	0.128 (0.102)

Significant levels p: * < 0.05, ** < 0.01, *** < 0.001,
