# Supplementary figures and images for: Functional Networks in Developmental Dyslexia: Auditory Discrimination of Words and Pseudowords
Source: NeuroSci. 2026 Feb 3;7(1):21. doi: 10.3390/neurosci7010021 (PMC12921797; doi:10.3390/neurosci7010021)

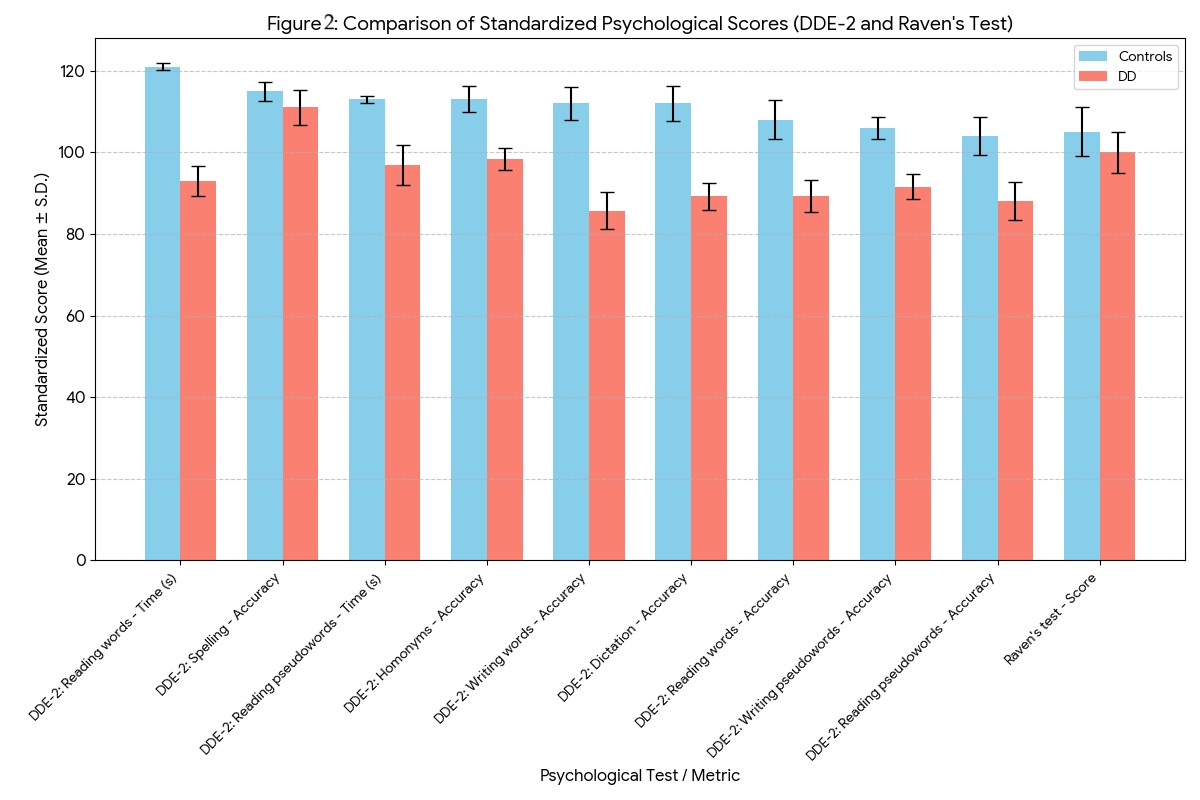

Supplement: Supplementary file 1 [file neurosci-07-00021-s001.zip › Supp_Figure S2_DD2_test.jpg]

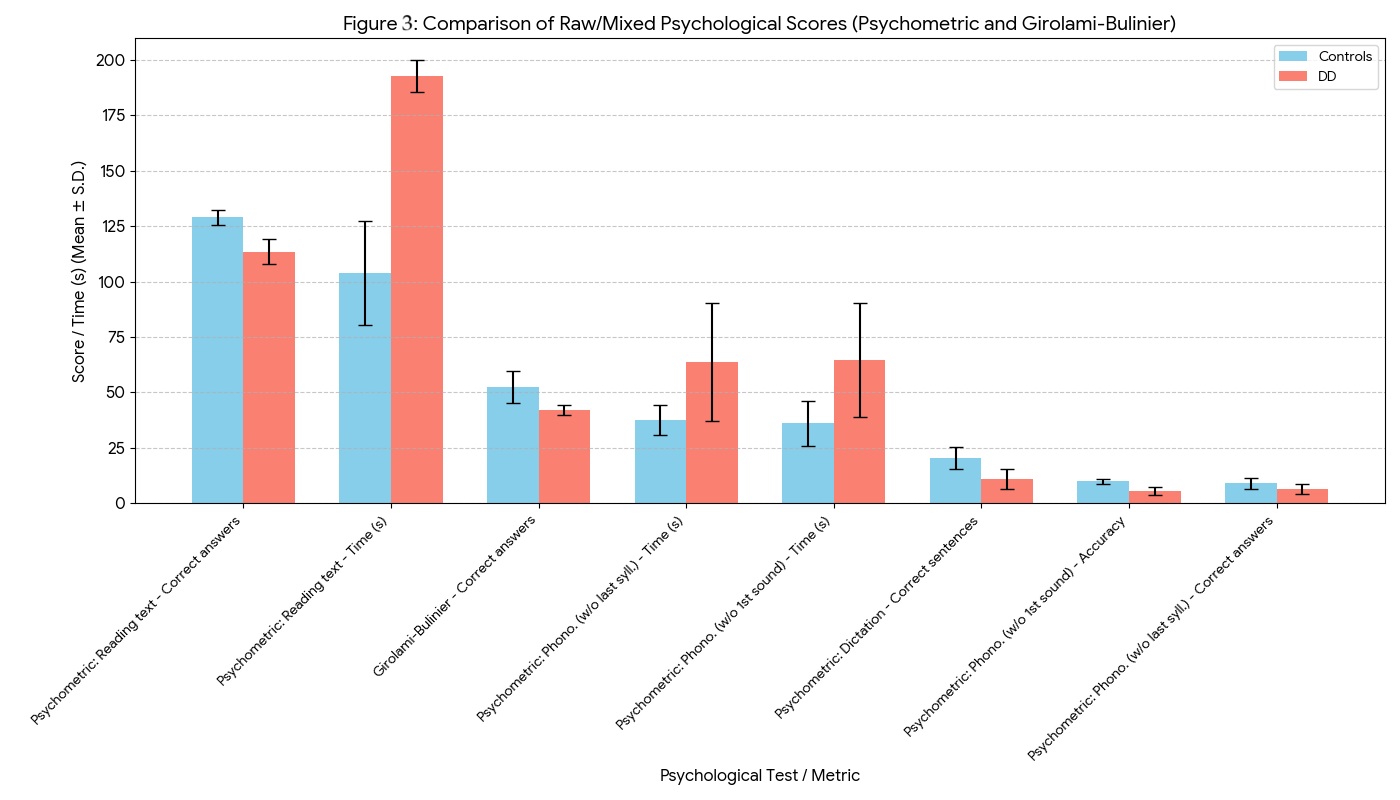

Supplement: Supplementary file 1 [file neurosci-07-00021-s001.zip › Supp_Figure S3_psychometric_test.jpg]

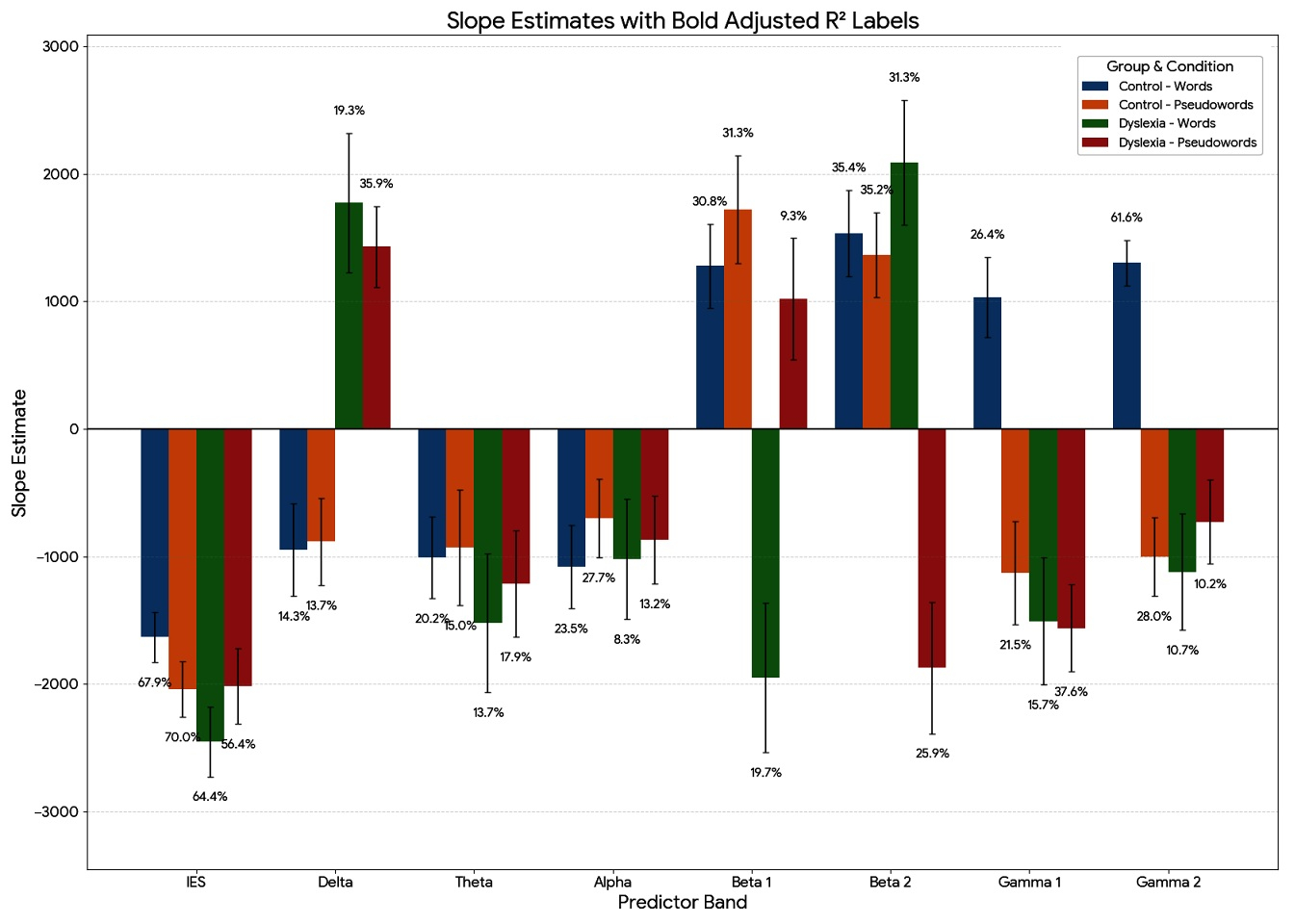

Supplement: Supplementary file 1 [file neurosci-07-00021-s001.zip › Supp_Figure_S4_summary_regression.jpg]

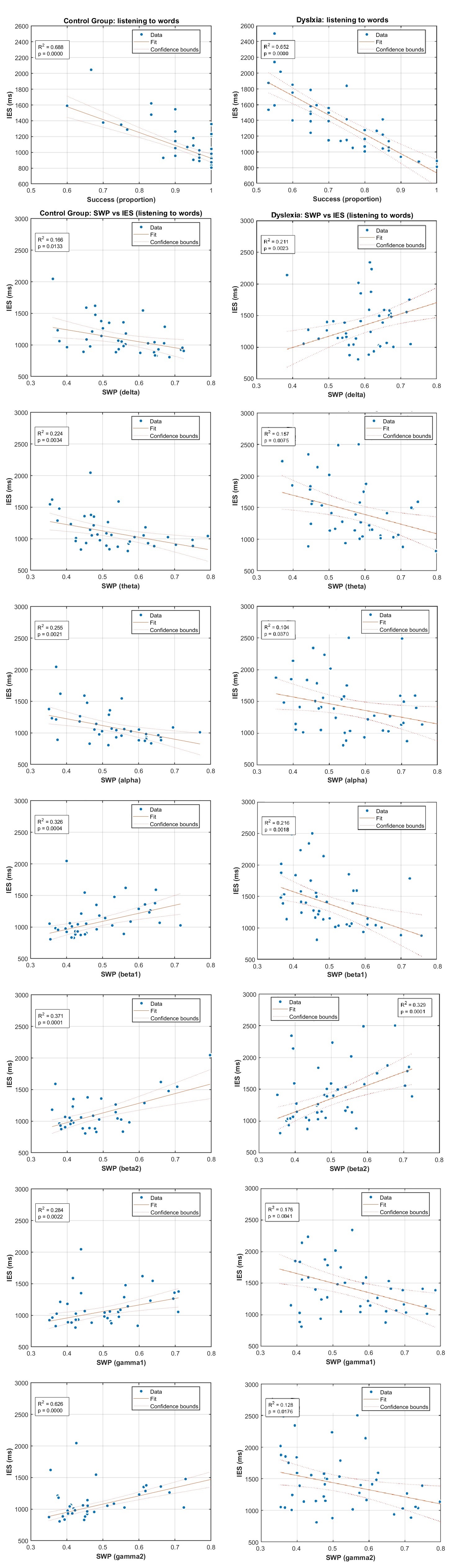

Supplement: Supplementary file 1 [file neurosci-07-00021-s001.zip › Supp_Figure_S5_regression_word_listening.jpg]

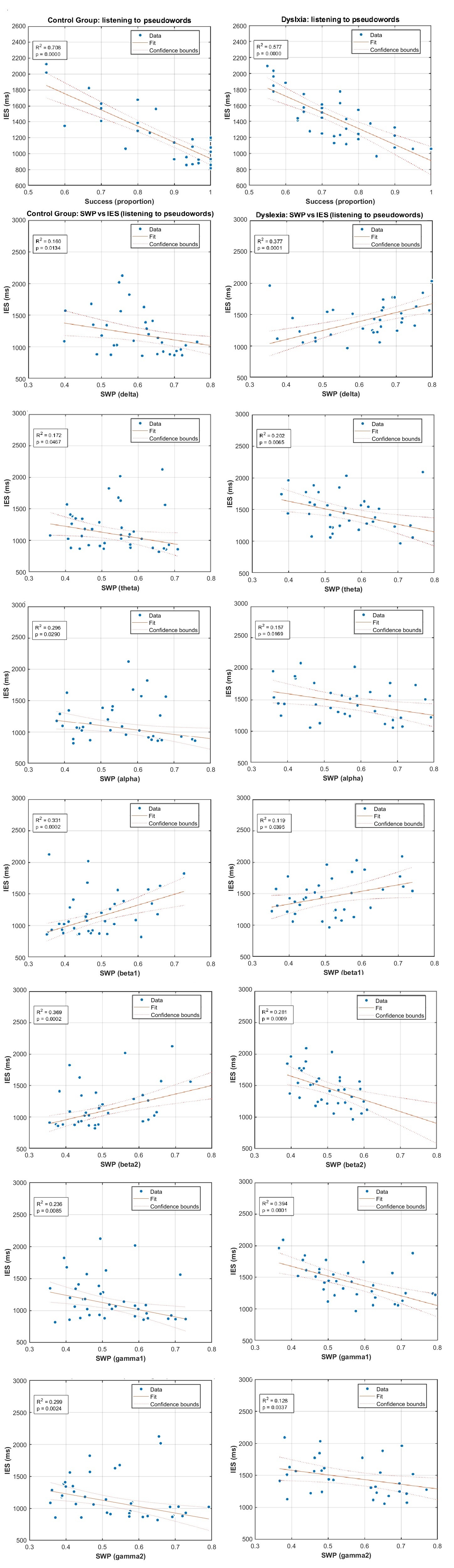

Supplement: Supplementary file 1 [file neurosci-07-00021-s001.zip › Supp_Figure_S6_regresion_pseudoword_listening1.jpg]
